# Whole genome transcriptional analysis of intestinal biopsies and blood cells indicate genes involved in antioxidant defense systems, amino acid metabolism and antigen presentation in the pathogenesis of celiac disease

**DOI:** 10.1186/s12916-025-04261-1

**Published:** 2025-08-29

**Authors:** Åsa Torinsson Naluai, Shafir Sabbag, Sanna Abrahamsson, Audur H. Gudjónsdóttir, Henrik Arnell, Daniel Agardh

**Affiliations:** 1 Department of Laboratory Medicine, Institute of Biomedicine, Sahlgrenska Academy at the University of Gothenburg, Gothenburg, Sweden; 2 Core Facilities, University of Gothenburg, Gothenburg, Sweden; 3 Pediatric Gastroenterology, Hepatology and Nutrition, Queen Silvia Children’s Hospital, Sahlgrenska University Hospital, Gothenburg, Sweden; 4 Pediatric Gastroenterology, Hepatology and Nutrition, Karolinska University Hospital and Women’s and Children’s Health, Karolinska Institute, Stockholm, Sweden; 5 Celiac disease and Diabetes Unit, Department of Clinical Sciences, Lund University, Malmö, Sweden

**Keywords:** Celiac disease, HLA, MHC, Autoimmunity, Oxidative stress, RNA sequencing, Small bowel, MTORC1, Mammalian target of rapamycin

## Abstract

**Background:**

Celiac disease is associated with HLA-risk haplotypes, but non-HLA genes and environmental factors are also linked to disease susceptibility. In this study, we explore the molecular pathways involved in celiac disease by analyzing the differential expression of genes in both the gut and peripheral blood across various celiac disease phenotypes.

**Methods:**

Whole genome RNA sequencing was performed on 283 samples from intestinal mucosa and peripheral blood from 72 cases with either active, potential, or treated celiac disease and 73 disease controls. Enrichr pathway analysis of top differentially expressed genes was performed.

**Results:**

Overall, 7565 genes in intestinal biopsies and 542 genes in blood samples were differentially expressed between cases and controls. Compared with controls, immunoglobulin heavy variable 5–51 (*IGHV5-51*) (*p* = 1.05 × 10^−14^) and tissue transglutaminase (*TGM2*) (*p* = 5.29 × 10^−10^), encoding for TG2, the main autoantigen in celiac disease, were two of the top up-regulated genes in intestinal biopsies from celiac cases. TGM2 was also slightly upregulated in blood cells from cases with active disease compared with controls (*p* = 0.05). The topmost differentially expressed genes in peripheral blood were *HLA-DQB1*, *HLA-DQB2*, and *GSTM1*. Among pathways identified containing transcriptionally differentiated genes were antioxidant defense systems (e.g., nuclear factor (erythroid-derived 2)-like 2 (Nrf2), glutathione, ergothioneine, and peroxisome metabolism), as well as MHC class 1 antigen presentation, amino acid transport, mTORC1, bilirubin and lipid metabolism, liver homeostasis, the complement system, and interferon signaling.

**Conclusions:**

Differentially expressed genes in cases and controls indicate crosstalk between molecular pathways involved in antioxidant defense, immune regulation, and nutrient signaling in the pathogenesis of celiac disease.

**Supplementary Information:**

The online version contains supplementary material available at 10.1186/s12916-025-04261-1.

## What is already known on this topic

Highly expressed genes can distinguish damaged intestinal mucosa present in celiac disease from normal healthy mucosa.

## What this study adds

This study presents RNA sequenced from several intestinal phenotypes such as from patients with damaged intestinal mucosa as well as from those without a damaged intestine but still an autoantibody response to tissue transglutaminase (“potential celiac disease”) or those with treated celiac disease. By combining phenotypes in a much larger number of tissue samples from both peripheral blood and mucosal biopsies, we found highly differentially expressed genes, but also identified genes involved in controlling and triggering celiac disease-associated changes.

## How this study might affect research, practice or policy

This study identifies molecular mechanisms involved in celiac disease and new possible targets for treatment and for identification of individuals at risk.

## Background

Genetic predisposition influences the risk for celiac disease (CD) and the most associated gene variants are located in the HLA region on chromosome six [1]. Another hallmark of CD is the presence of autoantibodies against the tissue transglutaminase 2 (TG2) protein, encoded by the gene *TGM2* [2].

Previously, a CD-specific transcriptomic signature was found to be associated with an increase of cell proliferation, nuclear division, and cell cycle activity [3]. Several studies have shown that some of the most highly expressed genes in CD mucosa are those encoding for immunoglobulin heavy variable 5–51 (*IGHV5-51*), C-X-C motif chemokine ligand 11 (*CXCL11*), interferon gamma (*IFNG*), and cytotoxic T-lymphocyte associated protein 4 (*CTLA4*) [4, 5]. Another study using single-cell transcriptomic analyses of the immune cell compartment from the intestine indicates that IFNG signaling might play a central role in the suppression of macrophage maturation and the accumulation of proinflammatory macrophages due to upregulation of the *IFI16* and *GBP2/4/5* genes and involvement of viral/bacterial-related pathways [6].

In the present study, these previous investigations were extended by using RNA sequencing in an unbiased analysis of all expressed genes to explore molecular changes occurring in children with different phenotypes of CD. Highly differentially expressed genes as well as perhaps the more important insights into disease mechanisms are likely to be gained from studying the duodenal mucosa. However, in this study, we extended previous in situ studies to also include samples from peripheral blood. We ranked the top differentially expressed genes in intestinal biopsies and in cells from blood using a combined analysis of different CD phenotypes.

## Methods

### Material

Enrolled were children referred to four pediatric clinics in Sweden for investigation with upper endoscopy and intestinal biopsy, as described previously [7]. Diagnosis of CD was based on the ESPGHAN criteria, i.e., an intestinal biopsy showing a Marsh (M) score ≥ 2 [8, 9]. In addition to multiple intestinal biopsies collected from the proximal duodenum and separately from the bulb for histopathological examinations, two separate duodenal biopsies were obtained for RNA extraction. For the purpose of clearly separating cases from controls, two children with M2 only were excluded from the analysis, resulting in a total of 72 children included as cases for the present study. Of those who were positive for TG2 autoantibodies at diagnosis, 48 children also had intestinal villous atrophy, i.e., active CD (a-CD), 18 children had normal intestinal mucosa findings, at the same time as they were positive for TG2 autoantibodies, i.e., potential CD (p-CD) and six children were examined for mucosal healing after treatment, i.e., treated CD (t-CD) (Table 1). Control patients were investigated for other diseases, but had normal intestinal histopathology, and were included as disease controls (Table 1). The vast majority of the disease controls were diagnosed with gastroenterological reflux disorder (GERD), recurrent abdominal pain (RAP), or Helicobacter pylori (HP) gastritis. Other diagnoses among disease controls were esophagitis, inflammatory bowel syndrome (IBS), wheat, and cow’s milk allergy. Children with Crohn’s disease were excluded from the analysis because of the possibility of having inflamed small intestinal mucosa. For the present study, a total of 283 patient samples passed quality control, and of these, 70 intestinal biopsies and 69 peripheral blood samples were from CD cases and 71 intestinal biopsies, and 73 peripheral blood samples were from disease controls, respectively.

**Table 1:** Characteristics of the patient material

	***Biopsy A-CD***	***Biopsy controls***	***Biopsy P-CD***	***Biopsy T-CD***	***Blood A-CD***	***Blood control***	***Blood P-CD***	***Blood T-CD***
N = 290*	52	70	18	6	47	71	18	8
mean age (SD)	7.72 (4.45)	11.22 (4.57)	7.01 (3.83)	11.74 (2.63)	7.40 (4.67)	11.16 (4.58)	7.09 (4.51)	9.06 (3.35)
Male sex (%)	20 (38.5)	31 (44.3)	8 (44.4)	2 (33.3)	18 (38.3)	30 (42.3)	9 (50.0)	3 (37.5)
Marsch score								
M0	-	54 (77.1)	11 (61.1)	1 (16.7)	-	57 (80.3)	13 (72.2)	2 (25.0)
M1	-	16 (22.9)	7 (38.9)	3 (50.0)	-	14 (19.7)	5 (27.8)	4 (50.0)
M2	3 (5.8) *	-	-	-	2 (4.3) *	-	-	-
M3	2 (3.8)	-	-	-	2 (4.3)	-	-	-
M3A	13 (25.0)	-	-	2 (33.3)*	16 (34.0)	-	-	2(25.0) *
M3B	23 (44.2)	-	-	-	15 (31.9)	-	-	-
M3C	10 (19.2)	-	-	-	11 (23.4)	-	-	-
M4	1 (1.9)	-	-	-	1 (2.1)	-	-	-
Control diagnoses								
GERD	-	23 (32.8)	-	-	-	24 (33.8)	-	-
RAP	-	11 (15.7)	-	-	-	10 (14.1)	-	-
Gastritis	-	7 (10.0)	-	-	-	9 (12.7)	-	-
H. pylori	-	6 (8.6)	-	-	-	7 (9.9)	-	-
IBS	-	6 (8.6)	-	-	-	3 (4.2)	-	-
U. colitis	-	4 (5.7)	-	-	-	5 (7.0)	-	-
Esophag	-	3 (4.3)	-	-	-	1 (1.4)	-	-
T1D	-	2 (2.9)	-	-	-	1 (1.4)	-	-
CMPA	-	1 (1.4)	-	-	-	1 (1.4)	-	-
Malabs	-	1 (1.4)	-	-	-	1 (1.4)	-	-
Other	-	6 (8.6)	-	-	-	9 (12.7)	-	-

^*^Seven samples excluded from analyses, resulting in a total of 283 samples

*Abbreviations*: *GERD* gastroenterological reflux disorder, *RAP* recurrent abdominal pain,* H. pylori* Helicobacter pylori, *U. colitis* Ulcerous colitis, *Esophag.* esophagitis, *T1D* type 1 diabetes, *CMPA* gastrointestinal diagnosis of cow’s milk protein allergy, Malabs.,,malabsorption. “Others” included heredity of CD, skin disorders, malabsorption, and wheat allergy

### RNA extraction and cDNA synthesis

Biopsy samples from patients undergoing small intestinal endoscopy were directly put in RNA later medium (Thermo Fisher Scientific Inc., CA, USA) and these were later processed for extraction and purification of total RNA using the Qiagen RNA kit or with the Maxwell 16 instrument (Promega, CA, USA) according to the manufacturer’s instructions. Blood samples were collected using Tempus tubes (Thermo Fisher Scientific Inc., CA, USA) and RNA from whole blood (i.e., all cells containing RNA) was extracted using the Maxwell 16 instrument (Promega, CA, USA).

### Whole genome RNA sequencing

The quality of the libraries was evaluated using the Fragment Analyzer (Advanced Analytical Technologies, Inc.) with the DNF-471 Standard Sensitivity RNA kit. Sequencing libraries were prepared from 10 to 500 ng total RNA using the Illumina Stranded Total RNA library preparation kit with Ribo-Zero Plus treatment (cat# 20,040,525/20040529, Illumina Inc. San Diego, CA). Unique dual indexes (cat# 20,040,553/20040554, Illumina Inc.) were used. The library preparation was performed according to the manufacturers’ protocol (# 1,000,000,124,514). The adapter-ligated fragments were quantified by qPCR using the Library Quantification kit for Illumina (KAPA Biosystems) on a CFX384 Touch instrument (Bio-Rad). Cluster generation and 150-cycle paired-end sequencing of the libraries were performed on the S4 flow cell using the NovaSeq 6000 system and v1.5 sequencing chemistry (Illumina Inc. San Diego, CA) at the SciLifeLab SNP&SEQ Technology Platform (Uppsala, Sweden). Demultiplexing and conversion to FASTQ format were done using the bcl2fastq2 (2.20.0.422) software provided by Illumina. Additional statistics on sequencing quality were compiled with an in-house script from the FASTQ files, RTA, and BCL2FASTQ2 output files. The RNA-seq data was analyzed using the best practice pipeline https://nf-co.re/rnaseq/3.3. In short, adapters and low-quality tails were trimmed from reads prior to read alignment. Clean sequence reads aligned to the human genome were used to assemble transcripts, estimate the abundance of these transcripts, quantify the genes, and detect differential expression among samples. For mRNA and lncRNA analyses, the reference genome build GRCh38/hg38 was chosen as the annotation references. Fragments per kilo-base million (FPKM) of both lncRNAs and mRNAs in each sample were calculated based on the length of the fragments and reads count mapped to this fragment.

### Differential expression analyses

**Fig. 1:**
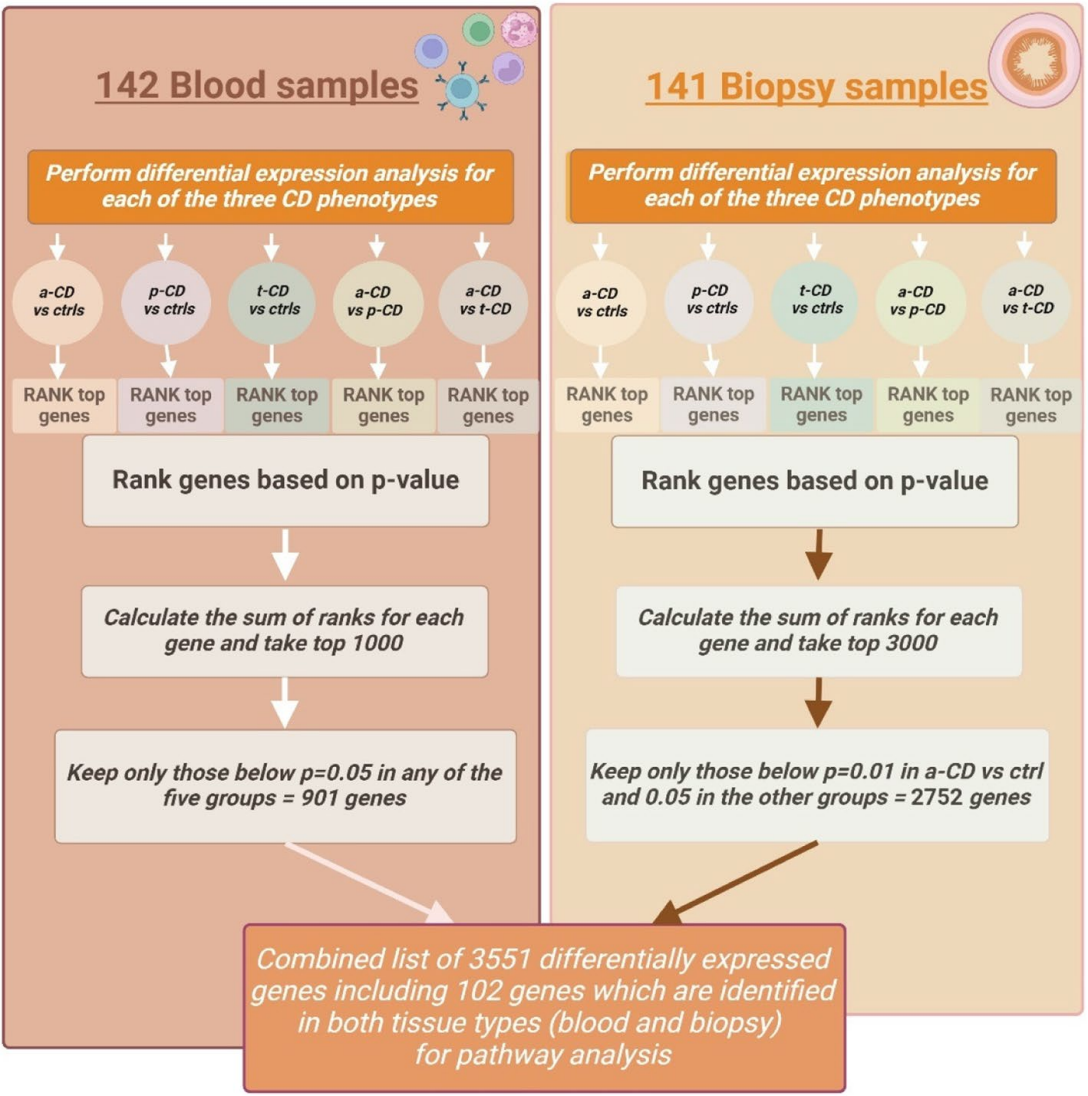
Flowchart showing the outline of the combined blood and biopsy analysis from patients with active celiac disease (a-CD), potential celiac disease (p-CD) and treated celiac disease (t-CD) compared with controls (ctrls) and the differential expression analysis resulting in 3551 genes for pathway analysis including 102 overlapping genes identified in both tissue types

**Fig. 2:**
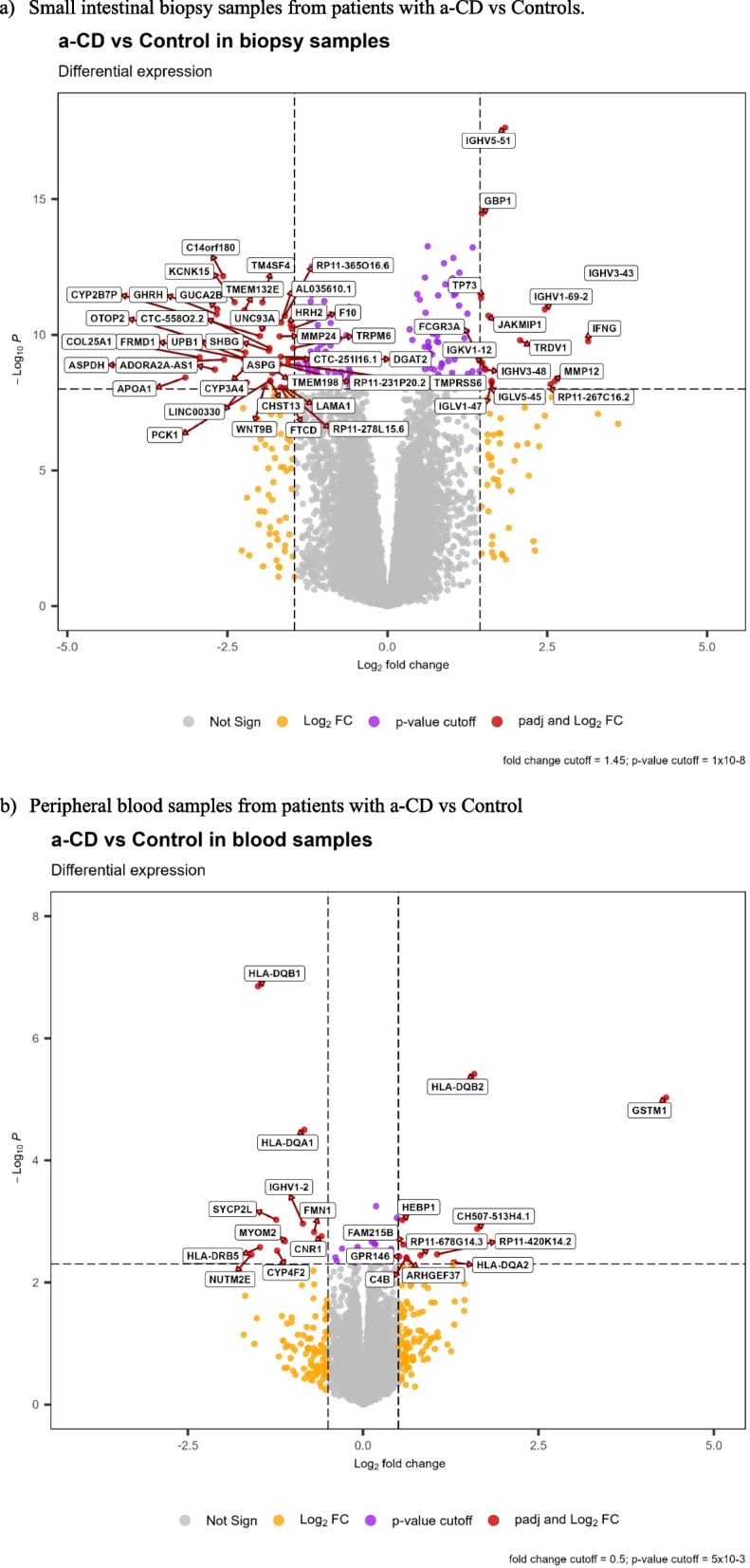
Volcano plot of differential gene expression in patients with active celiac disease (a-CD) compared with controls. **a** Small intestinal biopsy samples from patients with a-CD vs. controls. **b** Peripheral blood samples from patients with a-CD vs. control

Differential expression analyses were conducted using limma-voom and the edgeR package [10]. The mean–variance trend was converted by the voom function into weights and used in the analysis of log-transformed RNA-seq counts. Normalized intensities were also offset from zero before transforming to the log scale and estimating the mean–variance relationship empirically. The fitted model included age, sex, and parents’ origin of birth, divided into four regions: (1) Western Europe (69%); (2) Finland, Eastern Europe, and the Middle East (25%); (3) remaining parts of Asia (2%); and (4) Africa (4%), respectively. Blood and intestinal biopsy samples were analyzed separately for each phenotype comparison: (1) a-CD, (2) p-CD, (3) t-CD compared with controls, (4) a-CD compared with p-CD, and (5) a-CD compared with t-CD. Levels of tissue transglutaminase autoantibodies in each group are plotted in Additional file 2: Fig. S1. An outline of the analyses is shown (Fig. 1). *P*-values were adjusted using a Benjamini–Hochberg false discovery rate. Volcano plots (Fig. 2) were generated showing the top up- and down-regulated genes for biopsy and blood samples separately.

### Clustering methods

**Fig. 3:**
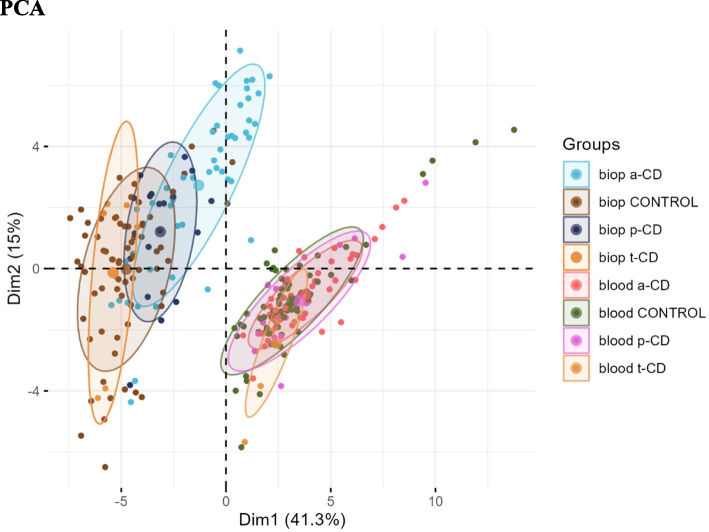
Principal component analysis (PCA) plot of small intestinal biopsies and peripheral blood samples of patients with active celiac disease (a-CD), potential celiac disease (p-CD) and treated CD (t-CD) compared with controls

**Fig. 4:**
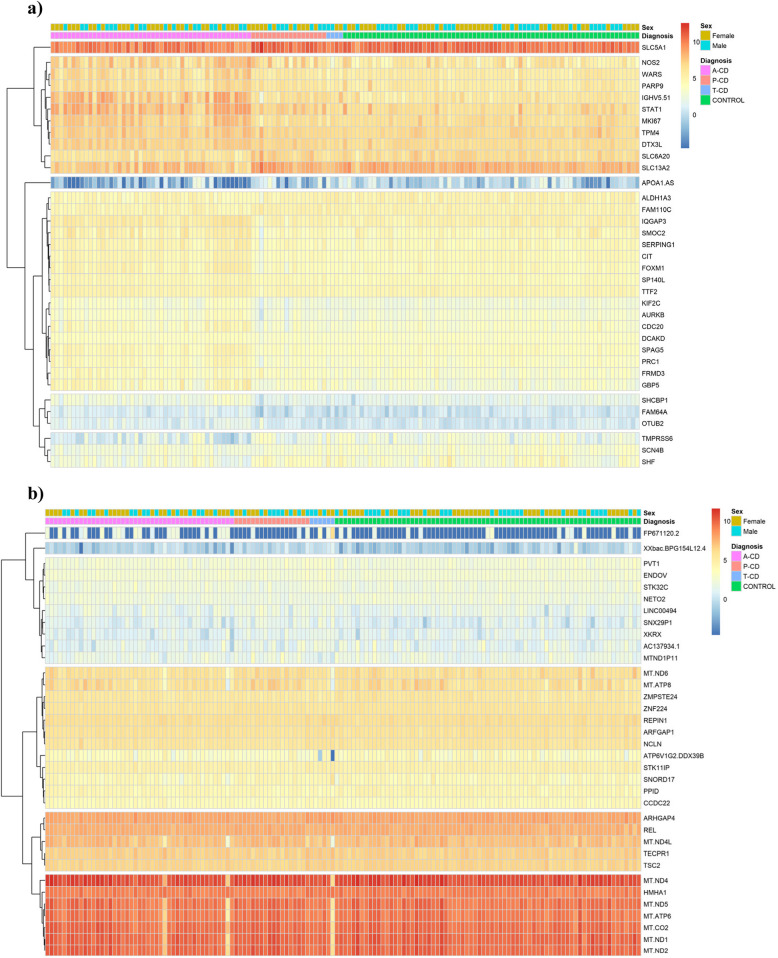
Heatmap of differential expressed genes of top 35 ranked genes in the combined analysis of each sample type, in active celiac disease (A-CD), potential celiac disease (P-CD) and treated celiac disease (T-CD) and controls, **a** intestinal biopsies and **b** peripheral blood

We used Deseq2 to perform two types of cluster analysis. First a principal component analysis (PCA) was performed, which identifies major sources of variation between samples [11] (Fig. 3). We also performed hierarchical clustering to visualize relationships between the most significant genes in each analysis of ranked genes in blood samples and ranked genes in biopsy samples using “pheatmap” in *R* [11] (Fig. 4).

### Pathway analysis

Pathway enrichment analysis using Enrichr [12–14] was first performed for all five different phenotype comparisons: (1) a-CD vs controls, (2) p-CD vs controls, (3) t-CD vs controls, (4) a-CD vs p-CD, and (5) a-CD vs t-CD. After that, we included all phenotypes in a combined gene list of the highest ranked differentially expressed genes. This list was generated by sorting the results in order by p-value and giving each gene a consecutive number for each of the five different phenotype comparisons, and for blood and biopsy samples separately. The sum of these five ranks (hereafter referred to as “SumRank”) was then calculated separately in each tissue. Finally, the top 1000 SumRank genes were selected from blood and the top 3000 SumRank from biopsies. Only those genes significant for at least one comparison of the five phenotype comparisons above (*p* < 0.01 for a-CD versus controls and *p* < 0.05 for the other four comparisons).

## Results

### Differential expression

Overall, 7565 genes were differentially expressed (nominally significant *p* < 0.05) in small intestinal biopsies between cases with a-CD and controls, and 5244 genes remained differentially expressed after adjusting for multiple comparisons. The top 30 most significant genes are presented in Table 2. For the analysis of peripheral whole blood samples and a-CD compared with controls, 542 genes were nominally significantly differentially expressed. Only HLA-DQB1, HLA-DQB2, and Glutathione S-transferase mu 1 (GSTM1) reached the adjusted significance level (Table 2). All results from expressed genes in biopsies are included in Additional file 1: Table S1 and for blood in Table S2.

**Table 2:**
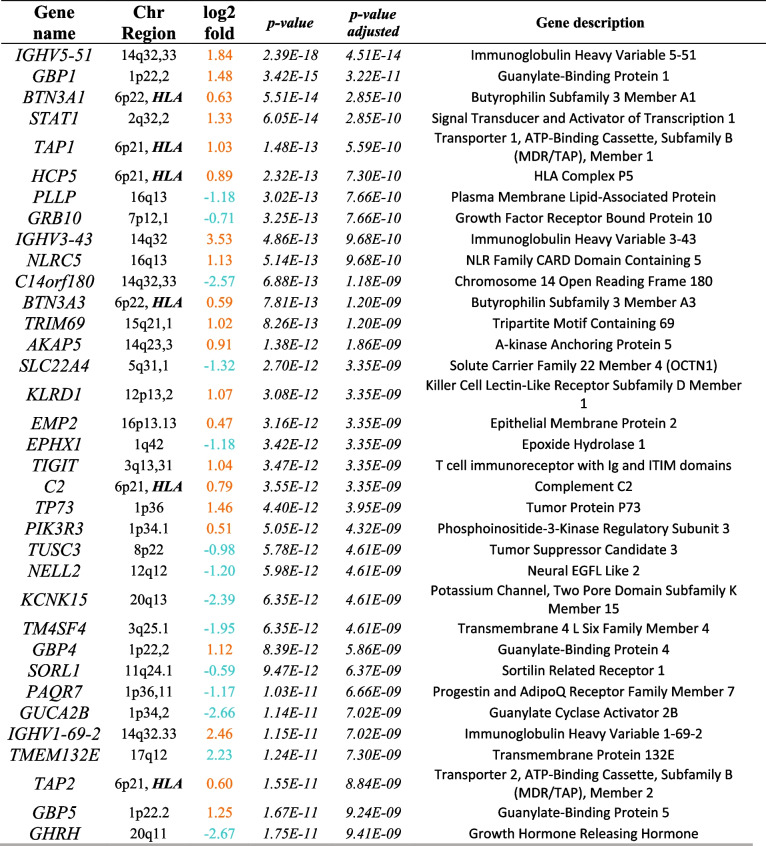

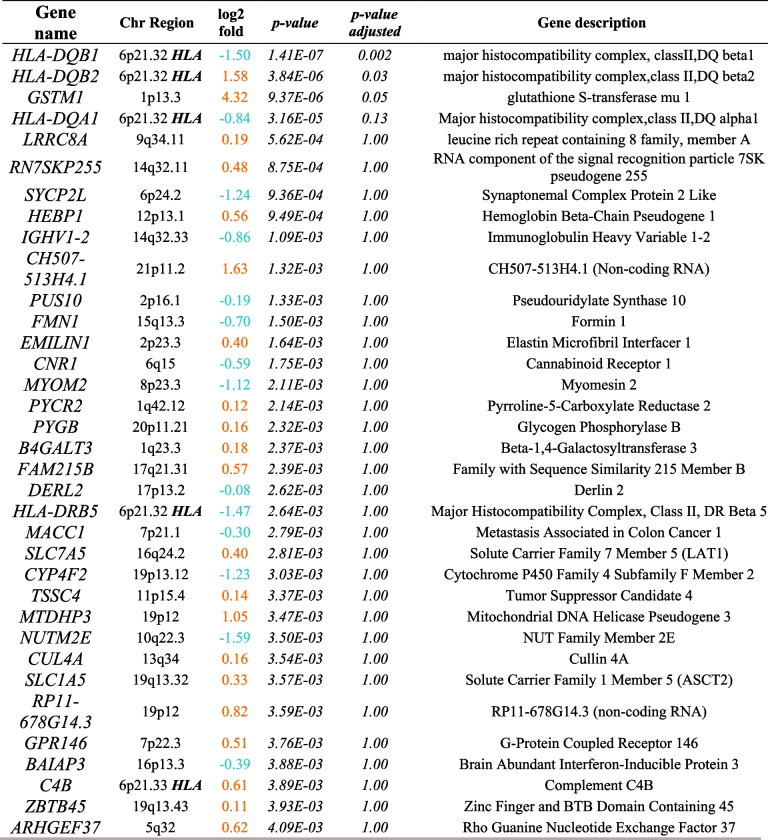
Top 35 most differentially expressed genes in intestinal biopsies of patients with active celiac disease (a-CD) compared with controls. Orange values represent an over expression while blue values represent a lower expression compared with controls

*log2fold = Log2 Fold Change*

**Table 3:**
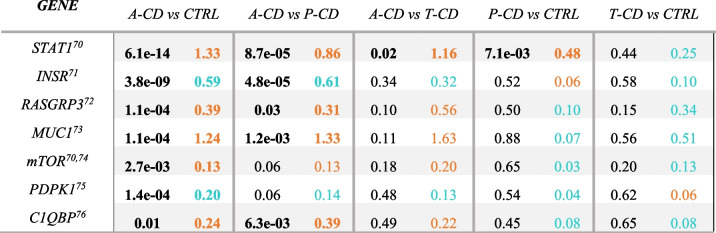
Top 35 most differentially expressed genes in peripheral blood samples from patients with active celiac disease (a-CD) compared with controls. Orange values represent an over expression while blue values represent a lower expression compared with controls

*log2fold = Log2 Fold Change*

### Differential expression in small intestinal biopsies 

Lipoprotein lipase (*LPL*) was the most upregulated gene in a-CD (log2 fold change (Log2FC) = 3.6, *p* = 1.9 × 10^−07^) (Fig. 2a). In cases with t-CD, compared with controls, the mitogen-activated protein kinase kinase kinase 20–antisense RNA 1 (MAP3K20-AS or MLK7-AS1) was upregulated almost 5 times (Log2FC = 4.79) and was the most significant gene (*p* = 2.32 × 10^−16^). This gene was also among the most upregulated in a-CD compared with controls (*p* = 8.31 × 10^−08^ and Log2FC = 3.30). In cases with p-CD versus controls, Ring finger protein 5 pseudogene 1 (*RNF5P1*) was the most significant gene (*p* = 4.08 × 10^−05^ and Log2FC = 2.65) and this gene was upregulated in both t-CD and p-CD, and significantly downregulated in a-CD. In cases with p-CD versus CD, gamma-aminobutyric acid receptor-associated protein like 1 (*GABARAPL1*) was the most significant and was upregulated (*p* = 1.61 × 10^−08^ and Log2FC = 0.91) (Table S2).

Of the top differentially expressed genes, *TGM2* was one of the most significantly up-regulated genes in intestinal biopsies in cases with a-CD compared to controls (Log2FC = 0.70, *p* = 5.29 × 10^−10^) as well as with p-CD compared to controls (Log2FC = 0.70) and a-CD compared to t-CD (Log2FC = 0.94). In contrast, protein kinase C delta (PKC-δ), encoded by the (*PRKCD*) a potential auto-antigen in CD [15] was significantly down-regulated in intestinal biopsies from cases with a-CD compared with controls. Several differentially expressed genes in this study interact with *PRKCD*, for example, *STAT1*, *INSR*, and *mTOR* (Table 3).

**Table 4:**
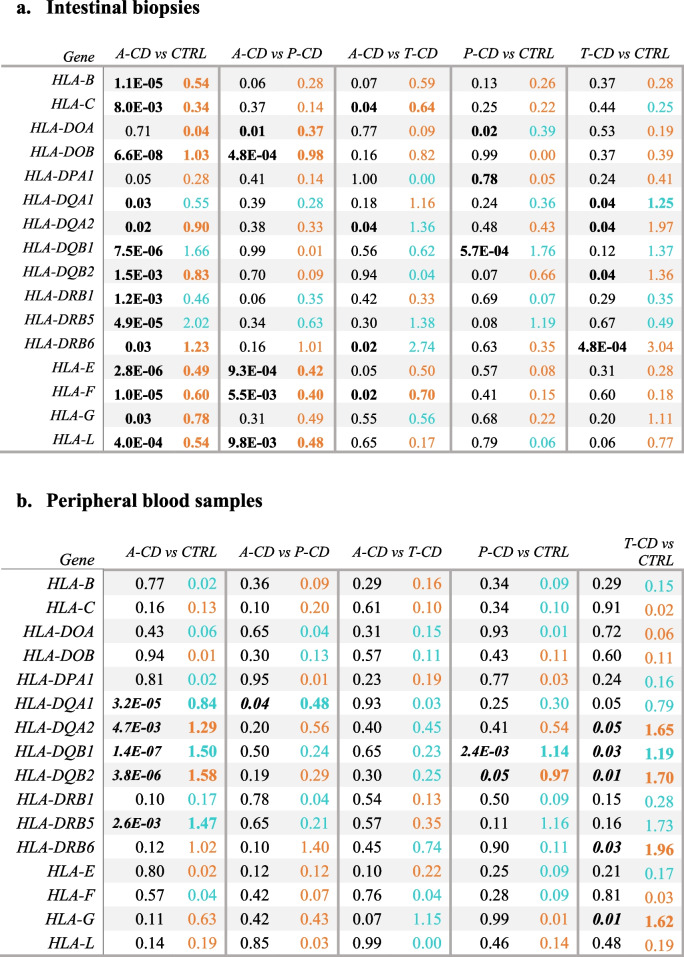
Differentially expressed genes in intestinal biopsies of patients with celiac disease (CD) compared with controls interacting with *PRKCD*. Orange values represent an over expression while blue values represent a lower expression compared with controls [16–22]

*log2fold = Log2 Fold Change*

The MHC genes, human leukocyte antigen class II, Beta 1, Beta 2, Alfa 1, and Alfa2 (*HLA-DQB1*, *HLA-DQB2*,* HLA-DQA1*, and *HLA-DQA2*), were all significantly expressed, of which the *DQB1* and *DQA1* genes were down-regulated and the *DQB2* and *DQA2* were up-regulated (Fig. 2a, Table 4). Expression of MHC class I and II genes is shown in Table 4.

**Table 5:**
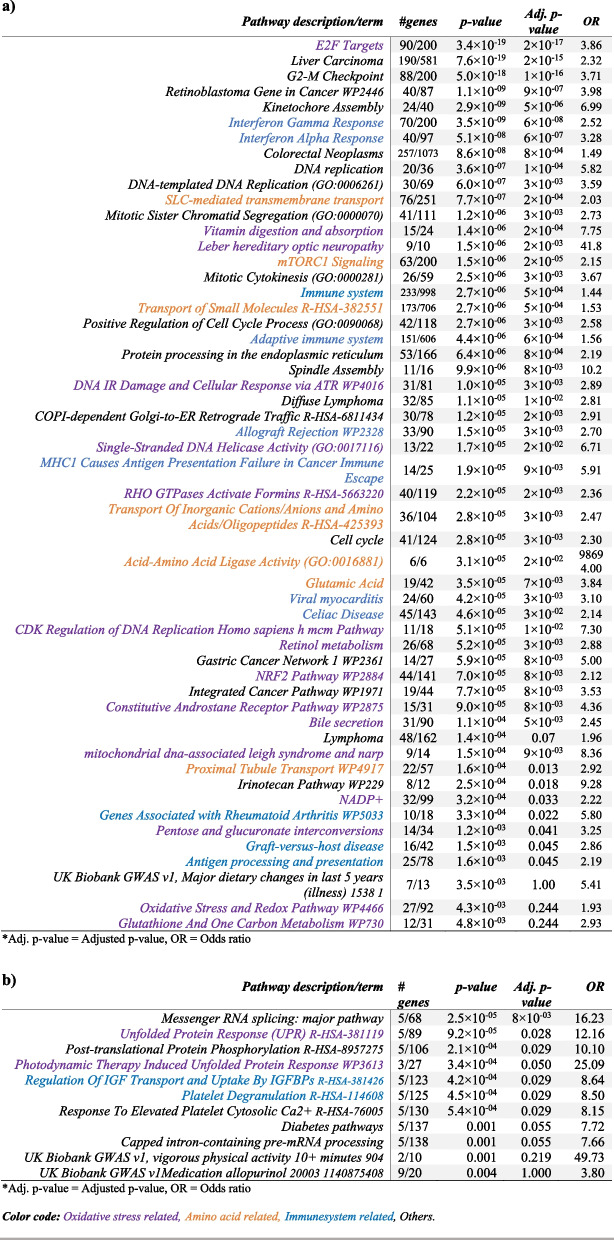
Differentially expressed *HLA* genes in intestinal biopsies (a) and peripheral blood samples (b) from patients with active celiac disease (a-CD), treated celiac disease (t-CD) and potential celiac disease (p-CD) compared with controls (ctrls). Orange values represent an over expression while blue values represent a lower expression compared with controls

*log2fold = Log2 Fold Change*

Highly up-regulated genes specifically in the a-CD compared with controls in small intestinal mucosa were antigen processing genes and transporters, “transporter 1, ATP-binding cassette, sub-family B” (*TAP1*) and “transporter 2, ATP-binding cassette, sub-family B” (*TAP2*). TAP1 was ranked 55 in the biopsy SumRank analysis. Other up-regulated genes involved in antigen presentation and located in the HLA region were Proteasome subunit beta type-8 and 9 (*PSMB8 *and* PSMB9*), the long non-coding RNA (lncRNA) named PSMB8 antisense RNA 1 (*PSMB8-AS1*) and the Butyrophilin subfamily 3 members A1 and A3 (BTN3A1, BTN3A2) and butyrophilin subfamily 3 member A2 (BTN2A2).

### Differential expression in whole blood

*GSTM1* was the most upregulated gene in peripheral blood from patients with a-CD, with a Log2FC of 4.32 and *p* = 9.37 × 10^−06^ (Fig. 2b, Table 2). *GSTM1* was also upregulated in intestinal biopsies in patients with CD (Log2FC = 2.31, *p* = 9.02 × 10^−03^). The RNA gene variant U1 small nuclear 18 (*RNVU1-18*) and mitochondrial ribosomal protein S31 (*MRM1*) reached adjusted significance in blood from t-CD compared with a-CD, Log2FC of 6.50, *p* = 1.34 × 10^−09^ and 0.55, *p* = 3.28 × 10^−06^, respectively. For t-CD compared with controls in blood samples, no gene reached the adjusted significance level; however, *RNVU1-18* was the most significant also here. The two most significant genes in the comparison of blood samples from p-CD and a-CD were two long noncoding (lnc) RNAs (*RP11-404F10.2* and *RP11-676B18.1*), which both reached the adjusted significance level, Log2FC of 0.82, *p* = 1.61 × 10^−07^ and 1.67, *p* = 2.74 × 10^−06^, respectively. In the comparison of blood samples from p-CD and controls, the genes lncRNA BAIAP2 antisense RNA 1 (*BAIAP2-AS1*) (Log2FC = − 1.13, *p* = 2.99 × 10^−04^ and complement factor 4 (*C4A*) (Log2FC = − 1.13, *p* = 1.08 × 10^−03^) were the most significant, although neither reaching adjusted significance.

### Differential expression in the combined phenotype analysis

**Fig. 5:**
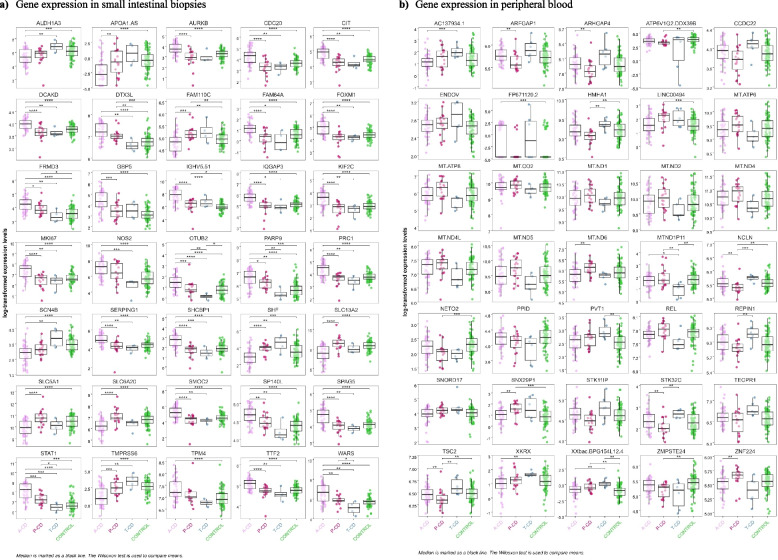
Box plot of the 35 top differentially expressed genes from the combined analysis in each sample type, adjusted for age, sex and parents’ origin of birth using the three celiac disease phenotypes compared with controls in **a** small intestinal biopsies and **b** peripheral blood samples. **a** Gene expression in small intestinal biopsies. **b** Gene expression in peripheral blood

The 35 top SumRanked genes in intestinal biopsies and the 35 top SumRanked genes in peripheral blood from each of the five combined phenotype analyses of a-CD, p-CD, t-CD, and controls are demonstrated in the heatmap (Fig. 4) and in box plots (Fig. 5). The top five genes identified by the SumRank for biopsy analysis were citron (*CIT*), tryptophanyl-tRNA synthetase (*WARS*), SHC binding protein 1 (*SHCBP1*), IQ motif containing GTPase activating protein 3 (*IQGAP3*), and the sodium-dependent proline transporter (*SLC6A20*) (Figs. 4 and 5). The top five genes identified by the SumRank for blood analysis were *TSBP1-AS1*, an antisense RNA transcript for the *TSBP1* (T-cell-specific basic protein 1) gene and for the *BTNL2* gene, as well as *MT-ND6*,* MT-ATP8*,* MT-ND5*, and *MT-ATP6*, which are all part of the mitochondrial genome and encode subunits of the mitochondrial electron transport chain and ATP synthase, which are essential for energy production within cells (Figs. 4 and 5).

**Fig. 6:**
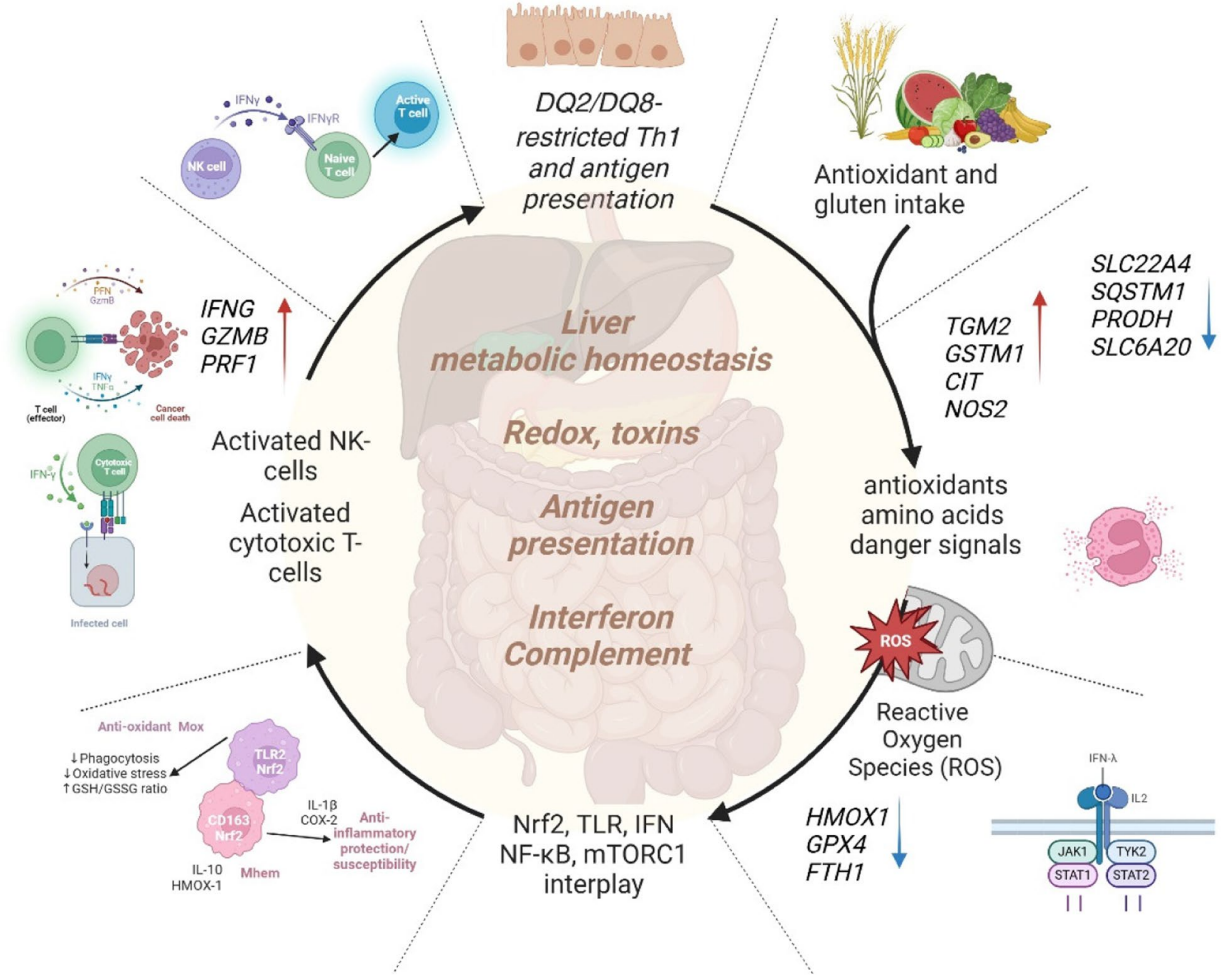
Overview of significant pathways. Among the pathways identified as transcriptionally differentiated were antioxidant defense systems (e.g., Nrf2, glutathione, ergothioneine and peroxisome metabolism), amino acid transport and mTORC1, bile secretion and liver homeostasis, antigen presentation, the complement system, and interferon signaling. Nrf2, nuclear factor erythroid 2 (NF-E2)-related factor 2; ROS, reactive oxygen species; mTORC1, mammalian target of rapamycin complex 1; TGM2, tissue transglutaminase (gene); HLA, human leucocyte antigen; NK cell, natural killer cell; SLC22A4, solute carrier family 22 member 4 (ergothioneine transporter); GSTM1, glutathione S-transferase mu 1; PRODH, proline dehydrogenase; SLC6A20, sodium-dependent proline transporter 1 (also ProT1). CIT encodes the citron protein. SQSTM1, sequestosome 1; NF-κB, nuclear factor kappa-light-chain-enhancer of activated B cells; HMOX1, haemoxygenase-1 (HO-1); FTH1, ferritin heavy chain 1; GPX4, glutathione peroxidase isoform 4; NOS2, nitric oxide synthase 2, inducible; GZMB, granzyme B; PRF1, perforin 1; IFNG, interferon gamma; created in BioRender (2024) https://BioRender.com

The Enrichr database was then used to analyze pathways belonging to each of these groups of differentially expressed genes (Table 5, Additional File 2 and 3: Table S2 and S3, Fig. 6).

## Enrichr pathway analysis of each phenotype comparison in intestinal biopsies

The Enrichr pathway analysis on differentially expressed genes, from intestinal biopsies in cases with a-CD compared with controls, among others demonstrated an aggregation of genes involving vitamin digestion and absorption, antigen processing and presentation, bile secretion, the nuclear factor erythroid 2 (*NF-E2*)-related factor 2 (Nrf2) pathway, interferon gamma and alpha signaling (*IFNG*), allograft rejection, retinol metabolism, amino acid transmembrane transporter activity, as well as metabolic pathway of high density and low density lipoproteins (HDL, LDL) (Additional file 2: Table S2).

## Enrichr pathway analysis of each phenotype comparison in blood samples

When comparing differentially expressed genes of blood samples in cases with a-CD with controls, pathways involving the cytosolic DNA-sensing pathway, steroid biosynthesis, insulin signaling, thiamine metabolism, peroxisome, glutathione binding, and metabolic reprogramming in pancreatic and colon cancer, among others, were identified. A specific pathway identified for the comparison between p-CD and a-CD, or controls was “taste transduction” including the taste receptor, type 2 (TAS2R), members 20, 31, 13, 14, and 19 (*TAS2R20*, *31*, *13*, *14* and *19*) (Additional file 2: Table S2, Fig. S2).

## Enrichr pathway analysis of the combined results from biopsy and blood samples

From the SumRank analysis, we merged the top genes from the blood (*n* = 1000) and from the biopsy analysis (*n* = 3000) where at least one of the five comparisons (1) a-CD vs controls, (2) p-CD vs controls, (3) t-CD vs controls, (4) a-CD vs p-CD, and (5) a-CD vs t-CD) had a *p*-value lower than 0.05 in all comparisons except for the comparison of a-CD versus controls in the biopsy samples, where 0.01 was used as a threshold. This procedure resulted in (*n* = 901) in blood and (*n* = 2752) in biopsy, with a total of 3551 unique genes, where 102 genes overlapped and were identified in both tissue types (Fig. 1). Differentially expressed genes in this combined biopsy and blood analysis were involved in toxins and antioxidant defense systems such as the Nrf2 and glutathione pathways, peroxisome, oxidoreductase activity, ABC-type xenobiotic transporter activity, constitutive androstane receptor (CAR) pathway, and bile secretion (Table 5, Additional file 3: Table S2). Pathways involving MHC class 1 antigen presentation failure, amino acid transport/mTORC1 signaling, as well as interferon alpha and gamma signaling, were also identified in this analysis (Table 5, Additional file 3: Table S3).

## Discussion

In our combined list of differentially expressed genes, some of the most differentially expressed genes in different phenotypes of CD compared with controls were those linked to pathways involving antigen processing and presentation, MHC class 1 deficiency, amino acid metabolism, lipid metabolism, and oxidative stress related pathways including the NRF2 pathway (WP2884), the oxidative stress and redox pathway (WP4466), retinol metabolism, bile secretion, and potent antioxidants such as ergothioneine and glutathione.

One of the most significant findings in patients a-CD compared with controls was the decreased gene expression of peroxisome proliferator-activated receptor gamma (*PPARG*), the gene encoding for the PPARγ protein. Increased oxidative stress downregulates PPARγ, which is believed to be mediated by TG2 in the intestinal mucosa [23]. The intestine as well as the liver are equipped with mechanisms to detoxify reactive intermediates and regulate oxidative stress as a defense against ingested toxins and pathogens. Nrf2 is a key regulator of reactive oxygen species (ROS) and the oxidative stress and oxidative phosphorylation (OXPHOS) response [24]. Interestingly, Nrf2 is also activated by energy-based signals (e.g., glucose fluctuations, calorie restriction) [25–27]. Recent work suggests that the Nrf2 transcription factor is critical for protecting the liver and gastrointestinal tract against disease by regulating a versatile cellular antioxidant defense [28, 29]. Increased ROS production activates Nrf2, which in turn affects metabolic reprogramming, glycolysis, and gluconeogenesis, a process integrating sequestosome 1 (SQSTM1) [30, 31], glycogen synthase kinase-3β (GSK-3β), mTORC1, and nuclear factor kappa-light-chain-enhancer of activated B cells (NF-κB), signaling [32]. Activation of Nrf2 inhibits NF-κB activation[33] and lead to transcriptional activation of major antioxidants and cytoprotective proteins such as heme oxygenase-1 (HO-1), encoded by the gene *HMOX1*, glutamate-cysteine ligase (GCL) encoded by the *GCLC* (the catalytic subunit) and *GCLM* (the modulatory subunit) genes, and the glutathione S-transferases (GSTs) family of genes, all of which help counteract oxidative stress and restore cellular homeostasis [34, 35]. HO-1 is a stress-responsive enzyme that degrades free heme to carbon monoxide (CO), iron, and biliverdin (converted into bilirubin) [36]. Nrf2 translocation and HO-1 expression/activity and the associated antioxidant response are important in antimicrobial processes and are also suggested to generally inhibit immune-mediated inflammatory diseases [36]. Glutathione and the glutathione peroxidases encoded by isoforms such as *GPX3* and *GPX4* are regulated by Nrf2 [37]. In this study, the glutathione S-transferase *GSTM1* is the most up-regulated gene in cells from blood and is also up-regulated in biopsies of patients with a-CD. *SQSTM1*, *HMOX1*, *GCLC*, *GCLM*, *GPX3*, and *GPX4* are all significantly down-regulated in biopsies from patients with a-CD compared with p-CD and controls. The liver is the main site of glutathione synthesis, exporting this peptide into both blood and bile [38]. CD is associated with a significant change in both cholesterol and bile metabolism, and it has previously been shown that a gluten-free diet decreases the level of cholesterol synthesis as well as the reabsorption of bile acids [39, 40]. An impaired redox balance and ROS have been shown to cause damage to different cell components, including proteins, lipids, and DNA. NADPH oxidases (NOX) are a group of different enzymes collectively involved in the production of ROS, in particular superoxide, which can create a respiratory burst, playing a crucial role in the innate immune response to pathogens and inflammatory and autoimmune diseases [41, 42]. NOX activity and expression have also been suggested to increase under gliadin-induced stress conditions [43]. Several additional identified pathways from the differentially expressed genes in the joint analysis of biopsy and blood were indirectly connected to oxidative stress. For example, the “CDK Regulation of DNA Replication Homo sapiens mcm Pathway” where pathogen-induced oxidative stress resulted in a strong up-regulation of these mini-chromosome maintenance factors (MCMs), a response completely abolished by treatment with glutathione [44]. Secondly, the constitutive androstane receptor (CAR) pathway (WP2875) is a nuclear receptor pathway that influences oxidative stress responses by modulating Nrf2 activity. Furthermore, the “Vitamin digestion and absorption pathway”—many vitamins, particularly vitamins C and E, help neutralize ROS and reduce oxidative stress. Proper absorption of these vitamins is crucial for maintaining the body’s antioxidant defense system [45, 46]. Finally, another example was the E2F targets pathway, where E2F1, a key member of the E2F family, has been shown to regulate intracellular ROS generation through various mechanisms, suggesting that E2F targets are involved in modulating cellular ROS levels as well as facilitating cell survival after oxidative stress [47–50].

Another finding related to the antioxidant defense system was the down-regulation of OCTN1 and ergothioneine (ET) transporter (ETT), encoded by the solute carrier family 22 member 4 (*SLC22A4*) [49] in CD cases compared with controls. ET is an intracellular antioxidant, which is absorbed from food, and the ETT is one of the identified genes implicated in autoimmunity [50]. ET is suggested to be a promising diet-derived cytoprotective agent that may promote longevity [51, 52]. Apart from being highly correlated to ET plasma levels, gene variants in *SLC22A4* are linked to a total of 67 traits reported in the GWAS catalog. Some of the top associations for this gene are myeloid white cell count, IBD, fatty acid measurement, coronary artery disease, hypercholesterolemia, asthma, and cardiovascular disease [53]. *SLC22A4* is located in the chromosome band 5q31, previously linked and associated with CD [54, 55].

Other identified pathways from our study involve amino acid transport and the mammalian target of rapamycin complex 1 (mTORC1) signaling. Metabolic pathways for nonessential amino acids such as proline are closely connected to the antioxidant and OXPHOS systems. For instance, the oxidation of proline by proline dehydrogenase (*PRODH*) leads to the formation of ROS [56]. Interestingly, proline catabolism is also connected to Nrf2, and together they are involved in regulating lipid metabolism during nutrient deprivation [57]. Also, genes involved in lipid and cholesterol metabolism show high differential expression in a-CD versus controls, such as the *LPL* and *APOA1* (apolipoprotein A1) and *APOB* (apolipoprotein B) genes, and several lipid pathways are among the most significant in the biopsy analysis; however, only in a-CD versus controls. One lipid pathway was significant in blood, and this was the cholesterol biosynthesis pathway WP197, which, in contrast, was found in the comparison between t-CD and controls. We have previously shown that the amino acid proline is increased in plasma from children with CD compared with controls [58]. In the present study, genes involved in proline metabolism such as *PRODH*, *GLS*, and *ALDH18A1* were down-regulated, whereas *ALDH4A1* was up-regulated in the gut of CD patients*.* Knockdown of aldh4a1 has been shown to induce the expression of Nrf2 targets in human 293 T cells [57]. Pyrroline-5-carboxylate reductase (*PYCR2*), which is one out of three isoforms that catalyzes the final step of proline biosynthesis, was up-regulated in cells from blood of a-CD patients compared with controls.

HO-1 may also regulate proline metabolism to control collagen synthesis, particularly in the context of tissue repair or fibrosis [56]. HO-1’s production of CO has anti-inflammatory effects by reducing the activity of pro-inflammatory cytokines, and overexpression of HO-1 decreases the lymphoproliferative response and differentiation of cytotoxic T cells [59] and protects against TNF-α-mediated airway inflammation [60]. Here, we observe a significant downregulation of the *HMOX1* gene, which encodes for HO-1, in a-CD compared to controls. Whether this reflects a reduction in protein concentration or indicates the presence of a negative feedback loop remains to be further investigated.

WARS is a stress-induced factor activated under oxidative stress, amino acid deprivation, or hypoxia. Solute carrier family 6 member 20 (*SLC6A20*), a sodium-dependent proline transporter responsible for the transport of proline across cell membranes, was in the top five sum ranked genes in the biopsy combined phenotype analysis. *SLC6A20* is crucial for the regulation of amino acid levels, particularly in tissues involved in protein metabolism and in the context of kidney function. Another transporter gene, *SLC5A1*, encoding for the sodium-glucose cotransporter 1 (SGLT1), was one of the top ranked genes in the biopsy analysis. This transporter plays a critical role in the absorption of glucose and galactose from the intestine, which is then transported into the bloodstream via GLUT2. SGLT1 inhibition can possibly prevent diabetes-induced ROS formation and glycogen accumulation [61].

The top SumRanked gene for the blood combined phenotype analysis was the transcription of *TSBP1-AS* (alias: *XXbac-BPG154L12.4*), located in the HLA-region. *TSBP1-AS* was upregulated in blood from both p-CD and t-CD compared with a-CD and controls. This gene has been shown to be triggered by TNF-α in in keratinocytes [62, 63]. and differential methylation was shown in patients with rheumatoid arthritis [64]. Gene variants at the *TSBP1-AS* locus are strongly associated with the levels of complement component 4 (C4), C2/LILRB4 protein level ratio, IGA glomerulonephritis, BMI, lipoprotein levels, and with many autoimmune diseases including celiac disease (GWAS catalog).

Of the most consistently up-regulated genes for all CD phenotypes were those of the antigen processing and presentation pathway, “transporter 1, ATP-binding cassette, sub-family B” (*TAP1*) and “transporter 2, ATP-binding cassette, sub-family B” (*TAP2*) both located in the HLA region. Higher gene expression levels of *TAP1* and *TAP2* may enhance the activation of antigen-specific T cells [65]. *TAP1*,* TAP2*,* PSMB8-AS1*, and *PSMB9* are all located in the HLA region and part of the antigen processing and presentation pathway. This pathway also includes the ER aminopeptidase encoded by *ERAP2* and the leukocyte immunoglobulin-like receptor subfamily B member 1 (*LILRB1*) gene, a gene associated with familiar autoimmunity [66]. *LILRB1* showed an increased expression in a-CD versus both controls and p-CD. *ERAP2* is upregulated in the gut of both a-CD and p-CD versus controls, and significantly downregulated in t-CD versus controls. *ERAP2* is reported to be associated with several other autoimmune diseases [67, 68] and is, as the TAP1 and TAP2 proteins, crucial for proper folding and assembly of the MHC-I heavy chain. Furthermore, a major regulator of MHC-I immune responses and expression is the *NLRC5* gene [69], which was one of the topmost upregulated genes in both a-CD and t-CD compared with controls. Following IFNγ stimulation*, NLRC5* is induced by activated *STAT1* (*IFNG* and *STAT1*, both among most upregulated genes in the gut of a-CD). The highest expression levels of *NLRC5* can be found in CD4 + T cells, CD8 + T cells, CD19 + B cells, natural killer (NK) cells and natural killer T (NKT) cells. *NLRC5* deficiency dramatically impairs normal expression of MHC class I, in T, NKT, and NK lymphocytes, and is therefore essential for efficient CD8( +) T cell responses [70]. We found even higher levels of *NLRC5* in t-CD than cases with a-CD, indicating a potential high default activation of CD8( +) T cells in patients with CD. *NLRC5* also upregulates expression of the MHC class I accessory genes *PSMB9* and *TAP1* [71]*.* CD8( +) T cell infiltration has been previously observed in all forms of CD-associated intestinal lesions as well as in p-CD cases [72]. These results are also aligned with a previous study demonstrating that gliadin can activate CD8( +) T lymphocytes [73] and that these cells are present in intestinal lesions from CD patients, indicating that class I antigen presentation may be linked to antigen-specific cytotoxic activity in the duodenal mucosa in a-CD.

We also identify differentially expressed genes coding for genes in the butyrophilin (BTN3 family) located in the HLA region and related to CD8( +) and gamma delta T cell responses. Butyrophilin subfamily 3 members A1 and A3 (*BTN3A1*,* BTN3A2*) and butyrophilin subfamily 3 member A2 (*BTN2A2*) are among the strongest upregulated genes in a-CD versus p-CD or controls. *BTN3A2* is also downregulated in a-CD and in t-CD versus controls in blood. Also, butyrophilin-like 3 and 8 (*BTNL3* and *BTNL8*) are downregulated in a-CD versus controls in biopsies. In previous studies, gluten triggered depletion of intraepithelial lymphocytes (IELs) with innate cytolytic properties and specificity for the *BTNL3* and *BTNL8*. The expression of *BTNL8* was reduced and these “natural” IELs were lost and replaced by IELs that failed to recognize *BTNL3/BTNL8* while producing *IFNG*. This change in IEL properties seemed to persist even after the gluten free diet [74].

As discussed above, several genes were differentially expressed in the HLA region. Some of these were also significant, or borderline significant, in the comparison between p-CD versus control or t-CD versus control, indicating a difference in individuals with both a-CD, p-CD, and t-CD versus controls. These genes were *PSMB9*, *C4B*, *TAP1*, *TAP2*, and *BTN3A2*. The same was also true for all the *HLADQA *and* DQB* genes in both blood and biopsy samples, with an even higher expression difference between either p-CD or t-CD with controls than with a-CD and controls (Table 4). Perhaps this could be an indicator of variation linked to the presence of different circulating immune cell populations of individuals with all phenotypes of celiac disease compared with controls and requires further investigation.

Additionally, we found that p-CD patients specifically had an increased expression of genes (taste 2 receptors, TAS2Rs) involved in bitter taste receptor activity (GO-0008527), Additional file 2: Table S2, Fig. S2). Previous studies have shown that inflammation has led to an increase in the expression of certain TAS2Rs in cells [75]. These bitter taste receptors can function as warning sensors and induce host defense mechanisms in the intestine. In human jejunal crypts, particularly those from individuals with obesity, these receptors induce the release of antimicrobial peptides and modulate the expression of innate immune factors (mucins, chemokines) and perhaps activate Nrf2-mediated oxidative stress [76]. Possibly, somehow these receptors can be part of what is keeping the integrity of the mucosa in these patients.

Finally, the study showed that the expression of *TGM2* was higher in both a-CD and t-CD, but not in cases with p-CD. One speculative explanation is that cells in these individuals have developed a mechanism to downregulate TGM2, thereby protecting the mucosa from the “mark” of autoantibodies and the subsequent damage. The present study also confirms that *IFNG*, *LPL*, *CTLA4*, *GBP1/2/4/5*, and *IGHV5-51* were highly up-regulated in the intestinal lesions from patients with a-CD. These genes are clearly part of defining the molecular profile of active CD.

A major strength of the present study is the large and well-characterized sample collection and the comparisons of expressed genes in both intestinal biopsies and peripheral blood. This multi-tissue gene expression profiling, including p-CD and t-CD patients, with apparently normal histologic features, allows for a more complex analysis. Expression in non-inflamed small intestinal regions in individuals still affected by the disease (t-CD) or producing antibodies without tissue damage (p-CD) might enhance the discovery of molecular pathways that may precede or underlie inflammation-driven tissue damage. This strategy is an attempt to try and distinguish between mechanisms which are the result of overwhelming expressional changes due to tissue damage (e.g., IFN signaling, and disruption of normal endothelial functions) from subtle, potentially causative mechanisms (e.g., antigen processing and presentation, mTORC1 and amino acid signaling, TAS2Rs and ROS/Oxidative stress pathway dysregulation), where these latter would not have been so easily identified without the multi-tissue, multi-phenotypic approach.

The study had several limitations. Firstly, we did not have enough biopsies with a Marsh score equal to 2 to evaluate potential differences in expression profiles between Marsh 2 and Marsh 3. We therefore chose not to stratify based on the severity of lesions due to this low number of samples categorized as Marsh 2. A second limitation was the lack of data on protein expression. RNA transcripts result in both regulatory RNA mechanisms and protein translation, and having data on protein levels to compare with could have significantly strengthened our findings. This will instead be an important aspect to include in future studies. Another limitation was the low number of t-CD patients.

## Conclusions

In summary, the current study presents analysis which bridges systemic inflammation (blood) and tissue-specific damage (biopsies), offering a more complete view of CD pathogenesis and potential candidate biomarkers for early intervention. Differentially expressed genes were involved in multiple interconnected pathways, indicating an intricate network of antigen presentation, redox regulation, and nutrient sensing mechanisms. Among others, the Nrf2, glutathione, ergothioneine, peroxisome, mTORC1, bitter taste receptor activity, MHC class I CD8( +) T cell responses, the complement system, and interferon signaling. Additionally, genes located in the HLA region, aside from *HLA-DQA1* and *HLA-DQB1*, were differentially expressed, suggesting a potential role for also these genes in the pathogenesis of CD. Functional experimental studies that intervene in these pathways are needed to explore the potential causal roles of these genes in CD pathogenesis.

## Supplementary Information

Additional File 1: Table S1. Results from all detected gene expression between phenotypes in biopsies (a) and in blood (b).

Additional File 2: Table S2, Fig. S1-S2, Table S2. The top 1000 differentially expressed genes in small intestinal biopsies between patients with active celiac disease and controls, (a-CD vs CTRL) and the top 500 genes in comparisons including potential CD and treated CD (a-CD vs p-CD, a-CD vs t-CD, p-CD vs CTRL and t-CD vs CTRL). Figure S1. Tissue transglutaminase auto-antibody levels in the different patient groups Figure S2. Bitter taste receptors (taste 2 receptors, TAS2Rs) levels in the different patient groups.

Additional File 3: Table S3. Table S3. Pathway analysis, using Enrichr, of all nominally significant pathways in the combined blood and biopsy analysis of 3551 genes (“all”) and in the overlapping 120 genes found in both tissue types (“overlap120”).

## Data Availability

Data availability Data is available on request from the European genome phenome archive (EGA). The accession numbers are; Study: EGAS50000001104, Dataset: EGAD50000001597, Experiment: EGAX50000039181.

## Abbreviations

CD: Celiac disease
a-CD: Active celiac disease
p-CD: Potential celiac disease
t-CD: Treated celiac disease
Nrf2: Nuclear factor erythroid 2 (NF-E2)-related factor 2 (protein)
ROS: Reactive oxygen species
mTORC1: Mammalian target of rapamycin complex 1 (protein)
TG2: Tissue transglutaminase 2 (protein)
*TGM2*: Tissue transglutaminase 2 (gene)
HLA: Human leucocyte antigen
MHC: Major histocompatibility complex
PKC-δ: Protein kinase C delta (protein)
*PRKCD*: Protein kinase C delta (gene)
