# Supplementary material for: Whole genome transcriptional analysis of intestinal biopsies and blood cells indicate genes involved in antioxidant defense systems, amino acid metabolism and antigen presentation in the pathogenesis of celiac disease
Source: BMC Med. 2025 Aug 29;23:507. doi: 10.1186/s12916-025-04261-1 (PMC12398127; doi:10.1186/s12916-025-04261-1)

**Table S2.** Pathway analysis, using Enrichr, and the top 1000 differentially expressed genes in small intestinal biopsies between patients with active celiac disease and controls, (a-CD vs CTRL) and the top 500 genes in comparisons including potential CD and treated CD (a-CD vs p-CD, a-CD vs t-CD, p-CD vs CTRL and t-CD vs CTRL).

| **BIOPSIES-PATHWAYS** | |  |  |  |  |  |
| --- | --- | --- | --- | --- | --- | --- |
| **Analysis** | **Pathway Term** | **number of genes** | **P-value** | **Adjusted P-value** | **Odds Ratio** | **Genes (The first 10 genes in each pathway are shown)** |
| *A-CD vs P-CD, A-CD vs CTRL* | Vitamin digestion and absorption | 10 | *1.2E-10* | *3.3E-08* | 28.41 | SCARB1;ABCC1;SLC46A1;BTD;FOLH1;TCN2;SLC23A1;SLC19A3;LRAT;APOB |
| *A-CD vs P-CD, A-CD vs CTRL* | Bile secretion | 16 | *7.2E-10* | *9.4E-08* | 8.68 | SCARB1;ABCC2;ABCB1;UGT1A1;EPHX1;ATP1A1;SLC5A1;CYP3A4;SLC4A4;NR0B2 |
| *A-CD vs P-CD, A-CD vs CTRL* | Retinol metabolism | 12 | *1.1E-07* | *9.3E-06* | 8.54 | SDR16C5;CYP2C9;UGT1A1;RETSAT;CYP2S1;UGT1A5;UGT1A4;LRAT;UGT1A3;CYP3A4 |
| *A-CD vs CTRL, A-CD vs T-CD, P-CD vs CTRL* | Antigen processing and presentation | 12 | *4.7E-04* | *1.7E-02* | 3.48 | IFNG;KLRC3;KLRC4;RFX5;TAP2;TAP1;KLRD1;HLA-DOB;CTSS;KIR2DL4;HLA-E;HLA-DQB1 |
| *A-CD vs P-CD, A-CD vs CTRL* | Nuclear Receptors Meta Pathway WP2882 | 29 | *1.6E-09* | *7.7E-07* | 4.15 | ABCB1;NR1I3;SLC5A11;TGFA;SLC5A1;SLC6A20;CYP3A4;CYP3A5;SLC2A7;SLC6A4;ME1;UGT |
| *A-CD vs P-CD, A-CD vs CTRL* | NRF2 Pathway WP2884 | 17 | *9.1E-08* | *1.1E-05* | 5.50 | SLC6A19;ABCC2;UGT1A1;SLC2A12;SLC6A13;SLC5A11;TGFA;SLC5A1;SLC6A20;PTGR1;SLC2 |
| *A-CD vs P-CD, A-CD vs CTRL* | Retinoic Acid | 9 | *3.0E-07* | *3.4E-05* | 12.31 | CYP2C9;UGT1A1;CYP2S1;UGT1A5;UGT1A4;UGT1A3;CYP3A4;CYP3A5;UGT2B7 |
| *A-CD vs T-CD* | Antigen Processing And Presentation Of Endogenous Peptide Antigen (GO:0002483) | 8 | *2.2E-08* | *4.1E-05* | 24.37 | ERAP2;HLA-B;TAP2;HLA-C;TAP1;HLA-F;HLA-E;ULBP3 |
| *A-CD vs CTRL, A-CD vs P-CD* | Mitotic Sister Chromatid Segregation (GO:0000070) | 22 | *2.6E-08* | *5.2E-05* | 4.78 | SPAG5;PLK1;NCAPG2;KIF14;CDCA8;NCAPG;KIF23;KIF11;SMC4;NCAPH |
| *A-CD vs CTRL* | Cholesterol Efflux (GO:0033344) | 10 | *5.9E-08* | *5.2E-05* | 14.75 | ABCG8;SCARB1;ABCG5;SOAT2;APOA1;APOC3;APOA4;ABCA8;TSKU;ABCG1 |
| *A-CD vs CTRL, A-CD vs P-CD* | Cholesterol Homeostasis (GO:0042632) | 17 | *6.7E-08* | *5.2E-05* | 5.96 | SCARB1;ABCG8;GRAMD1B;ABCG5;DGAT2;EPHX2;IL18;APOA1;APOC3;NR1H3 |
| *A-CD vs CTRL, A-CD vs P-CD* | Positive Regulation Of Cell Cycle Process (GO:0090068) | 22 | *8.1E-08* | *5.2E-05* | 4.43 | GPSM2;EDN1;EDN3;SPAG5;EGF;INSR;NCAPG2;KIF14;CDCA8;NCAPG |
| *A-CD vs CTRL, A-CD vs P-CD* | Sterol Homeostasis (GO:0055092) | 17 | *8.3E-08* | *5.2E-05* | 5.85 | SCARB1;ABCG8;GRAMD1B;ABCG5;DGAT2;EPHX2;IL18;APOA1;APOC3;NR1H3 |
| *A-CD vs CTRL* | Cholesterol Transport (GO:0030301) | 14 | *1.3E-07* | *6.6E-05* | 7.28 | SCARB1;ABCG8;ABCG5;STARD5;APOA1;APOC3;APOA4;ABCA8;TSKU;LIPC |
| *A-CD vs P-CD* | Positive Regulation Of Cell Cycle Process (GO:0090068) | 14 | *1.5E-06* | *1.7E-03* | 5.37 | EDN3;SPAG5;INSR;NCAPG2;CDCA8;TGFA;NCAPH;AURKB;CDC25B;CIT |
| *A-CD vs CTRL* | High-Density Lipoprotein Particle Remodeling (GO:0034375) | 7 | *3.5E-06* | *1.5E-03* | 16.74 | SCARB1;LIPC;LIPG;APOA1;APOC3;APOA4;ABCG1 |
| *A-CD vs P-CD* | Nitrogen Compound Transport (GO:0071705) | 16 | *3.5E-06* | *2.6E-03* | 4.35 | SLC22A4;SLC6A19;ABCC1;SLC22A5;ABCC2;SLC6A13;SLC6A12;SLC3A1;SLC1A4;SLC6A20 |
| *A-CD vs CTRL* | Cholesterol Transfer Activity (GO:0120020) | 8 | *1.1E-06* | *5.6E-04* | 15.31 | ABCG8;GRAMD1B;ABCG5;STARD5;APOA1;APOA4;APOB;ABCG1 |
| *A-CD vs CTRL* | Sterol Transfer Activity (GO:0120015) | 8 | *1.8E-06* | *5.6E-04* | 13.92 | ABCG8;GRAMD1B;ABCG5;STARD5;APOA1;APOA4;APOB;ABCG1 |
| *A-CD vs CTRL* | Carbohydrate:Monoatomic Cation Symporter Activity (GO:0005402) | 6 | *2.7E-06* | *5.8E-04* | 28.67 | SLC2A9;SLC23A2;SLC5A9;SLC5A11;SLC5A1;SLC5A2 |
| *A-CD vs CTRL, A-CD vs P-CD* | Carboxylic Acid Transmembrane Transporter Activity (GO:0046943) | 13 | *5.4E-06* | *8.5E-04* | 5.43 | SLC22A4;SLC23A2;SLC46A1;ABCC1;SLC6A19;SLC6A14;SLC6A13;SLC6A12;SLC1A4;SLC6A20 |
| *A-CD vs P-CD* | Amino Acid Transmembrane Transporter Activity (GO:0015171) | 8 | *7.1E-05* | *1.1E-02* | 6.59 | SLC22A4;SLC6A19;SLC6A13;SLC6A12;SLC3A1;SLC1A4;SLC6A20;SLC25A44 |
| *A-CD vs P-CD* | ABC-type Xenobiotic Transporter Activity (GO:0008559) | 4 | *7.2E-05* | *1.1E-02* | 26.20 | ABCC1;ABCB1;ABCC2;ABCG2 |
| *A-CD vs CTRL* | Sterol Binding (GO:0032934) | 11 | *1.4E-04* | *1.6E-02* | 4.39 | SULT2B1;GRAMD1B;STARD5;SOAT2;OSBPL3;RORC;APOA1;APOC3;NR1H3;ABCG1 |
| *A-CD vs CTRL* | Cholesterol Binding (GO:0015485) | 10 | *1.5E-04* | *1.6E-02* | 4.79 | SULT2B1;GRAMD1B;STARD5;SOAT2;OSBPL3;APOA1;APOC3;NR1H3;ABCG1;OSBP2 |
| *A-CD vs P-CD* | Solute:Sodium Symporter Activity (GO:0015370) | 6 | *1.8E-04* | *2.0E-02* | 8.45 | SLC5A11;SLC5A1;SLC6A20;SLC4A4;SLC28A1;SLC17A4 |
| *A-CD vs P-CD* | Glucose Transmembrane Transporter Activity (GO:0005355) | 5 | *2.7E-04* | *2.0E-02* | 10.36 | SLC2A9;SLC2A12;SLC5A11;SLC5A1;SLC2A7 |
|  |  |  |  |  |  |  |
| **BLOOD-PATHWAYS** | |  |  |  |  |  |
| **Analysis** | **Pathway Term** | **number of genes** | **P-value** | **Adjusted P-value** | **Odds Ratio** | **Genes (The first 10 genes in each pathway are shown)** |
| *A-CD vs T-CD* | Cytosolic DNA-sensing pathway | 7 | *9.7E-04* | *2.4E-01* | 4.93 | TBK1;IRF3;POLR1C;RIPK1;POLR3H;POLR2H;POLR2K |
| *T-CD vs CTRL* | Steroid biosynthesis | 4 | *1.4E-03* | *2.9E-01* | 9.82 | SQLE;NSDHL;DHCR7;FDFT1 |
| *A-CD vs T-CD* | Homologous recombination | 5 | *3.4E-03* | *4.2E-01* | 5.46 | RAD52;RAD51;RBBP8;BRCC3;NBN |
| *A-CD vs T-CD* | Other types of O-glycan biosynthesis | 5 | *6.2E-03* | *4.3E-01* | 4.68 | C1GALT1C1;GALNT1;MFNG;C1GALT1;GXYLT1 |
| *A-CD vs T-CD* | RNA polymerase | 4 | *7.1E-03* | *4.3E-01* | 5.82 | POLR1C;POLR3H;POLR2H;POLR2K |
| *T-CD vs CTRL* | Thiamine metabolism | 3 | *5.6E-03* | *6.1E-01* | 9.80 | NFS1;ALPL;AK4 |
| *A-CD vs T-CD* | Peroxisome | 6 | *1.7E-02* | *6.8E-01* | 3.10 | ABCD4;IDH1;AGPS;MPV17;ACSL3;ACOX3 |
| *A-CD vs T-CD* | Sulfur relay system | 2 | *1.6E-02* | *6.8E-01* | 13.05 | CTU2;CTU1 |
| *P-CD vs CTRL* | Lysosome | 7 | *4.2E-02* | *1.0E+00* | 2.27 | LAPTM4B;ATP6V0B;HEXB;FUCA2;CTSW;PPT2;CTSD |
| *P-CD vs CTRL* | Protein processing in endoplasmic reticulum | 10 | *1.1E-02* | *1.0E+00* | 2.45 | EDEM3;VCP;SEC13;RPN1;RRBP1;SEC61B;MAN1B1;P4HB;SEC23B;SEC24C |
| *P-CD vs CTRL* | Taste transduction | 6 | *2.1E-02* | *1.0E+00* | 2.95 | TAS2R20;TAS2R31;TAS2R13;TAS2R46;TAS2R14;TAS2R19 |
| *A-CD vs CTRL* | Insulin Signaling WP481 | 12 | *7.1E-04* | *6.6E-02* | 3.22 | RPS6KA4;MAP2K3;XBP1;PFKL;RPS6KA5;MYO1C;MINK1;SLC2A1;PIK3R2;SGK3 |
| *A-CD vs CTRL* | Metabolic Reprogramming In Pancreatic Cancer WP5220 | 6 | *5.8E-04* | *6.6E-02* | 6.57 | GPI;SLC7A5;PFKL;SLC2A1;PGM3;SLC1A5 |
| *A-CD vs CTRL* | N Glycan Biosynthesis WP5153 | 7 | *5.2E-04* | *6.6E-02* | 5.52 | GPI;B4GALT3;DPM2;ALG6;SRD5A3;MGAT4B;ALG11 |
| *A-CD vs CTRL* | Nucleotide Excision Repair in Xeroderma Pigmentosum WP5114 | 8 | *5.6E-04* | *6.6E-02* | 4.72 | DDB1;CUL4A;CDK7;SLX4;GPS1;POLD1;RPA1;RAD23A |
| *A-CD vs CTRL* | Nucleotide Excision Repair WP4753 | 6 | *7.5E-04* | *6.6E-02* | 6.22 | CUL4A;DDB1;CDK7;POLD1;RPA1;RAD23A |
| *A-CD vs CTRL* | FOXA2 Pathway WP5066 | 4 | *1.6E-03* | *1.2E-01* | 9.24 | SREBF1;GSTM2;SLC27A5;PPARGC1B |
| *T-CD vs CTRL* | Cholesterol Biosynthesis Pathway WP197 | 4 | *4.2E-04* | *1.5E-01* | 14.29 | SQLE;NSDHL;DHCR7;FDFT1 |
| *A-CD vs CTRL, A-CD vs T-CD* | Glycosylation And Related Congenital Defects WP4521 | 4 | *3.2E-03* | *2.0E-01* | 7.48 | DPM2;ALG6;SRD5A3;ALG11 |
| *A-CD vs T-CD* | Cytosolic DNA Sensing Pathway WP4655 | 8 | *5.1E-04* | *2.1E-01* | 4.79 | TBK1;IRF3;TRADD;POLR1C;RIPK1;POLR3H;POLR2H;POLR2K |
| *A-CD vs CTRL, A-CD vs P-CD* | Metabolic Reprogramming in Colon Cancer WP4290 | 5 | *3.8E-03* | *2.1E-01* | 5.31 | GPI;PFKL;SLC2A1;PYCR2;SLC1A5 |
| *T-CD vs CTRL* | trans,trans-Farnesyl Diphosphate | 3 | *7.9E-04* | *4.0E-02* | 23.54 | FNTB;DHDDS;FDFT1 |
| *A-CD vs T-CD* | 11-cis-Retinol | 2 | *5.9E-03* | *1.9E-01* | 26.10 | RDH11;RDH5 |
| *A-CD vs T-CD* | tRNA Modification (GO:0006400) | 10 | *6.0E-06* | *1.1E-02* | 6.96 | DUS2;CTU2;CTU1;ANKRD16;SSB;PUS1;WDR4;TPRKB;TRMT1;TRMT44 |
| *P-CD vs CTRL, A-CD vs P-CD* | Bitter Taste Receptor Activity (GO:0033038) | 6 | *3.6E-05* | *1.2E-02* | 11.83 | TAS2R20;TAS2R31;TAS2R46;TAS2R13;TAS2R14;TAS2R19 |
| *P-CD vs CTRL, A-CD vs P-CD* | Taste Receptor Activity (GO:0008527) | 6 | *1.0E-04* | *1.7E-02* | 9.46 | TAS2R20;TAS2R31;TAS2R46;TAS2R13;TAS2R14;TAS2R19 |
| *A-CD vs CTRL* | Glutathione Binding (GO:0043295) | 4 | *4.4E-05* | *1.7E-02* | 31.44 | GSTM4;GSTM2;GSTM1;PTGES2 |
|  |  |  |  |  |  |  |
| **BIOPSIES-DISEASES and GWAS** | |  |  |  |  |  |
| **Analysis** | **Pathway Term** | **number of genes** | **P-value** | **Adjusted P-value** | **Odds Ratio** | **Genes (The first 10 genes in each pathway are shown)** |
| *A-CD vs CTRL, A-CD vs P-CD* | Liver Carcinoma | 94 | *2.1E-24* | *3.1E-21* | 3.94 | ARHGAP11A;TOP2A;VIPR1;KDM8;HJURP;KIF14;BUB1B;HSPB1;MCM10;FASLG |
| *A-CD vs P-CD, A-CD vs CTRL* | Serum Metabolite Levels | 31 | *1.5E-11* | *3.1E-08* | 4.83 | SLC22A4;PHLPP2;SCARB1;ENPEP;SLC22A5;TREH;PIK3R3;SLC1A4;HYKK;UGT1A5 |
| *A-CD vs P-CD, A-CD vs CTRL* | Hartnup disease | 6 | *1.9E-07* | *1.1E-04* | 39.46 | SLC15A1;SLC6A19;RNF128;SLC3A1;RNF186;SLC6A20 |
| *A-CD vs CTRL, A-CD vs P-CD* | Colorectal Neoplasms | 92 | *2.6E-07* | *1.6E-03* | 1.86 | TOP2A;ABCD2;SERPINE2;DSCC1;BUB1B;SAT2;FASLG;ADM;BRCA1;FOXM1 |
| *A-CD vs CTRL* | Celiac Disease | 23 | *6.7E-07* | *5.0E-04* | 3.70 | GBP5;CXCL9;ACE;TNFRSF9;SLC6A14;LPL;APOC3;UPB1;MMP12;CXCL11 |
| *A-CD vs CTRL* | Apo A-I deficiency | 33 | *9.4E-07* | *1.4E-03* | 2.81 | SCARB1;GALT;SERPINE2;LPL;BRCA1;ACAT1;LIPC;CA2;SMPD1;LIPG |
| *A-CD vs CTRL* | Liver Cirrhosis, Experimental | 72 | *1.0E-06* | *3.2E-03* | 1.94 | VIPR1;SLC46A1;CXCL9;CERK;GPR68;WIPF1;TUSC3;SLC23A1;AQP7;ADM |
| *A-CD vs CTRL* | Kidney Failure, Chronic | 46 | *1.6E-06* | *3.4E-03* | 2.29 | SCARB1;SLC46A1;WIPF1;LPL;PDXP;ADM;ADRA1B;CYP3A4;CTSS;ACAT1 |
| *A-CD vs CTRL* | Cholesteryl Ester Transfer Protein Deficiency | 8 | *2.8E-06* | *3.7E-03* | 12.76 | SCARB1;LIPC;LIPG;APOA1;APOC3;LPL;APOA4;APOB |
| *A-CD vs CTRL* | Mammary Neoplasms, Human | 48 | *3.2E-06* | *3.7E-03* | 2.19 | BCAR3;TOP2A;GRB7;DNMT1;CXCL9;SHMT1;LEF1;HMMR;BRCA1;FOXM1 |
| *A-CD vs CTRL* | Lymphoproliferative Disorders | 28 | *3.5E-06* | *3.7E-03* | 2.90 | CD274;DNMT1;IGHV4_34;LPL;FASLG;PRDM1;TNFSF13B;TRGC1;FCGR3A;CASP8 |
| *A-CD vs P-CD* | Histidine Betaine (Hercynine) Levels | 5 | *3.9E-06* | *2.7E-03* | 32.82 | SLC22A4;UGT1A1;UGT1A5;UGT1A4;UGT1A3 |
| *A-CD vs CTRL* | Hyperlipoproteinemia type 3 | 18 | *4.3E-06* | *4.3E-03* | 3.98 | SCARB1;ABCG5;ACE;GALT;DGAT1;NOS2;EGF;LPL;APOA1;APOC3 |
| *T-CD vs CTRL* | Methemoglobinemia | 4 | *5.6E-06* | *5.0E-03* | 78.62 | CYB5R3;HBG2;HBB;HBA1 |
| *A-CD vs P-CD* | Xanthurenate Levels | 5 | *6.6E-06* | *2.7E-03* | 28.13 | UGT1A1;UGT1A5;UGT1A4;UGT1A3;SLC17A4 |
| *A-CD vs P-CD* | Urinary Metabolite Levels in Chronic Kidney Disease | 11 | *7.6E-06* | *2.7E-03* | 5.99 | SHF;AOC1;FOLH1;ACOX1;SLC6A13;DPEP1;ACADM;UNC93A;GTSE1;CYP3A5 |
| *A-CD vs CTRL* | Atherosclerosis | 90 | *8.6E-06* | *7.8E-03* | 1.70 | SCARB1;CXCL9;ITGB5;PRF1;HSPB1;FASLG;ADM;BRCA1;PTPRF;CTSS |
| *A-CD vs P-CD* | Severe diarrhea | 6 | *9.3E-06* | *1.8E-02* | 15.78 | ABCC2;UGT1A1;SLC5A1;CYP3A4;SLC6A4;ABCG2 |
| *A-CD vs CTRL* | Congenital aplastic anemia | 18 | *9.5E-06* | *5.6E-03* | 3.73 | FANCI;TOP2A;MDC1;BLM;STAT1;XRCC2;PLK1;BRCA1;MSH6;HEBP1 |
| *A-CD vs P-CD* | Drug-Induced Liver Disease | 25 | *1.0E-05* | *1.8E-02* | 2.86 | HCP5;ABCB1;NR1I3;CYP3A4;HRH2;LRAT;ABCC1;DGAT2;ABCC2;MLKL |
| *A-CD vs CTRL* | Hyperlipoproteinemia type 2 | 18 | *1.1E-05* | *5.6E-03* | 3.69 | SCARB1;ABCG8;ABCG5;ACE;NOS2;EGF;LPL;APOA1;APOC3;CYP3A4 |
| *A-CD vs CTRL* | Hereditary fructose intolerance | 21 | *1.1E-05* | *5.6E-03* | 3.27 | RYR2;BLM;G6PC;ACE;GALT;HGD;LPL;HSPB1;NR1H3;CYP3A4 |
| *A-CD vs CTRL* | Hepatitis C | 67 | *1.2E-05* | *9.9E-03* | 1.83 | TOP2A;SCARB1;VIPR1;CXCL9;SETD7;PRF1;FASLG;MKI67;ZFYVE1;CTSS |
| *A-CD vs P-CD* | Sitosterolemia | 9 | *1.7E-05* | *1.7E-02* | 6.99 | ABCC1;ABCC2;ABCB1;SOAT2;NPC1L1;APOB;TRPM6;ABCG1;ABCG2 |
| *A-CD vs CTRL* | Insulin-like growth factor 1 resistance to | 18 | *1.8E-05* | *7.6E-03* | 3.54 | ACE;IGFBP5;EGF;STAT1;IGFBP3;INSR;LPL;BRCA1;FBXO32;C2;HEBP1 |
| *A-CD vs CTRL* | Hyperlipoproteinemia | 8 | *1.9E-05* | *9.5E-03* | 9.01 | ABCG8;ABCG5;LIPC;EPHX2;LPL;APOC3;APOA4;APOB |
| *A-CD vs P-CD* | Crigler Najjar syndrome type 1 | 8 | *1.9E-05* | *1.7E-02* | 8.11 | ABCC2;UGT1A1;UGT1A4;UGT1A3;CYP3A4;CYP3A5;UGT2B7;C2 |
| *A-CD vs CTRL* | Fanconi anemia | 17 | *2.2E-05* | *7.6E-03* | 3.63 | FANCI;TOP2A;MDC1;BLM;STAT1;XRCC2;PLK1;BRCA1;MSH6;HEBP1 |
| *A-CD vs CTRL* | Myelodysplasia | 13 | *3.2E-05* | *1.2E-02* | 4.46 | FANCI;GINS1;XRCC2;BUB1B;BRCA1;RAD51;DKC1;FANCD2;RFWD3;TRIP13 |
| *A-CD vs P-CD* | Post-traumatic Stress Disorder (Re-Experiencing Symptoms) | 5 | *3.3E-05* | *2.7E-03* | 17.90 | MSRA;NBEA;MYZAP;CYP3A4;GCOM1 |
| *A-CD vs P-CD* | Metabolic Traits | 8 | *4.1E-05* | *2.7E-03* | 7.19 | SLC22A4;SLC2A9;ENPEP;UGT1A5;UGT1A4;UGT1A3;APOB;KLKB1 |
| *A-CD vs P-CD* | Crigler-Najjar Syndrome | 4 | *4.4E-05* | *1.5E-02* | 31.44 | UGT1A1;UGT1A5;UGT1A4;UGT1A3 |
| *A-CD vs P-CD* | Lucey-Driscoll Syndrome | 4 | *4.4E-05* | *1.5E-02* | 31.44 | UGT1A1;UGT1A5;UGT1A4;UGT1A3 |
| *A-CD vs P-CD* | Aldosterone Levels | 4 | *4.4E-05* | *2.7E-03* | 31.44 | UGT1A1;UGT1A5;UGT1A4;UGT1A3 |
| *A-CD vs P-CD* | Bilirubin Degradation Product, C17H20N2O5 (1) Levels | 4 | *4.4E-05* | *2.7E-03* | 31.44 | UGT1A1;UGT1A5;UGT1A4;UGT1A3 |
| *A-CD vs P-CD* | Bilirubin Levels In Extreme Obesity | 4 | *4.4E-05* | *2.7E-03* | 31.44 | UGT1A1;UGT1A5;UGT1A4;UGT1A3 |
| *A-CD vs P-CD* | Biliverdin Levels In Chronic Kidney Disease | 4 | *4.4E-05* | *2.7E-03* | 31.44 | UGT1A1;UGT1A5;UGT1A4;UGT1A3 |
| *A-CD vs P-CD* | Gilbert Disease | 4 | *7.2E-05* | *1.8E-02* | 26.20 | UGT1A1;UGT1A5;UGT1A4;UGT1A3 |
| *A-CD vs CTRL* | Marfan Syndrome | 48 | *7.7E-05* | *2.0E-02* | 1.90 | BCAR3;TOP2A;GRB7;DNMT1;CXCL9;SHMT1;LEF1;HMMR;BRCA1;FOXM1 |
| *A-CD vs CTRL* | Myelodysplastic Syndrome | 16 | *8.1E-05* | *2.0E-02* | 3.38 | FANCI;GINS1;BLM;XRCC2;IDH2;BUB1B;BRCA1;RAD51;FANCD2;DKC1;RFWD3;TRIP13;NAGS |
| *A-CD vs CTRL, A-CD vs P-CD* | Lactose intolerance | 4 | *8.6E-05* | *6.1E-02* | 38.15 | TREH;SLC5A1;ALDOB;LCT |
| *A-CD vs CTRL* | Human immunodeficiency virus infectious disease | 12 | *1.4E-04* | *6.1E-02* | 4.04 | FCGR3A;FCGR3B;IFNG;ATAD2;CD38;GZMB;FASLG;CACNA2D4;CYP3A4;SAMHD1 |
| *A-CD vs P-CD* | Bilirubin metabolic disorder | 6 | *2.4E-04* | *5.4E-02* | 7.88 | ABCC2;UGT1A1;NR1I3;UGT1A5;UGT1A4;UGT1A3 |
| *A-CD vs P-CD* | Neutropenia | 8 | *4.2E-04* | *5.9E-02* | 4.94 | SLC13A2;G6PC;ABCC2;ABCB1;UGT1A1;PLK1;CYP3A4;AURKB |
|  |  |  |  |  |  |  |
| **BLOOD-DISEASES and GWAS** | |  |  |  |  |  |
| **Analysis** | **Pathway Term** | **number of genes** | **P-value** | **Adjusted P-value** | **Odds Ratio** | **Genes (The first 10 genes in each pathway are shown)** |
| T-CD vs CTRL | Methemoglobinemia | 4 | *5.6E-06* | *5.0E-03* | 78.62 | CYB5R3;HBG2;HBB;HBA1 |
| P-CD vs CTRL | Leigh disease | 8 | *2.9E-06* | *1.3E-03* | 10.92 | MT-ND6;MTFMT;SURF1;MT-ND5;NDUFA2;NDUFAF5;MT-ND2;MT-ND1 |
| P-CD vs CTRL, A-CD vs T-CD | Leber hereditary optic neuropathy | 4 | *7.2E-05* | *1.6E-02* | 26.20 | MT-ND6;MT-ND5;MT-ND2;MT-ND1 |
| A-CD vs CTRL | Congenital hemolytic anemia | 6 | *8.4E-05* | *4.2E-02* | 9.86 | GPI;EPB42;SLC2A1;SLC4A1;SPTB;KLF1 |
| A-CD vs CTRL | Dehydrated hereditary stomatocytosis | 6 | *1.7E-05* | *4.0E-02* | 13.92 | GPI;EPB42;SRC;STOM;SLC4A1;SPTB |
| A-CD vs CTRL | Mean corpuscular volume 30040 raw | 33 | *2.8E-05* | *1.2E-02* | 2.33 | NOTCH3;ALAS2;FOCAD;DOCK3;YTHDC1;LY75;APOLD1;ANKRD9;SENP7;STK11 |
| A-CD vs CTRL | Mean reticulocyte volume 30260 raw | 26 | *4.2E-04* | *4.3E-02* | 2.19 | DOCK3;PIGQ;LY75;SLC2A1;PTPRF;SCAMP5;ANKRD9;HEBP1;CA1;SKP2 |
| A-CD vs CTRL | Mean sphered cell volume 30270 raw | 27 | *3.8E-04* | *4.3E-02* | 2.17 | NOTCH3;PIGQ;LY75;SLC2A1;EFCAB7;MEOX1;APOLD1;ANKRD9;CA1;SKP2 |
| A-CD vs CTRL | Red blood cell distribution width 30070 raw | 20 | *3.1E-04* | *4.3E-02* | 2.55 | KANK2;RBM38;ALAS2;CCDC176;LY86;SLC2A1;SLC4A1;ERAL1;SPTB;SELENBP1 |

**Figure S1.** Tissue transglutaminase auto-antibody levels in the different patient groups


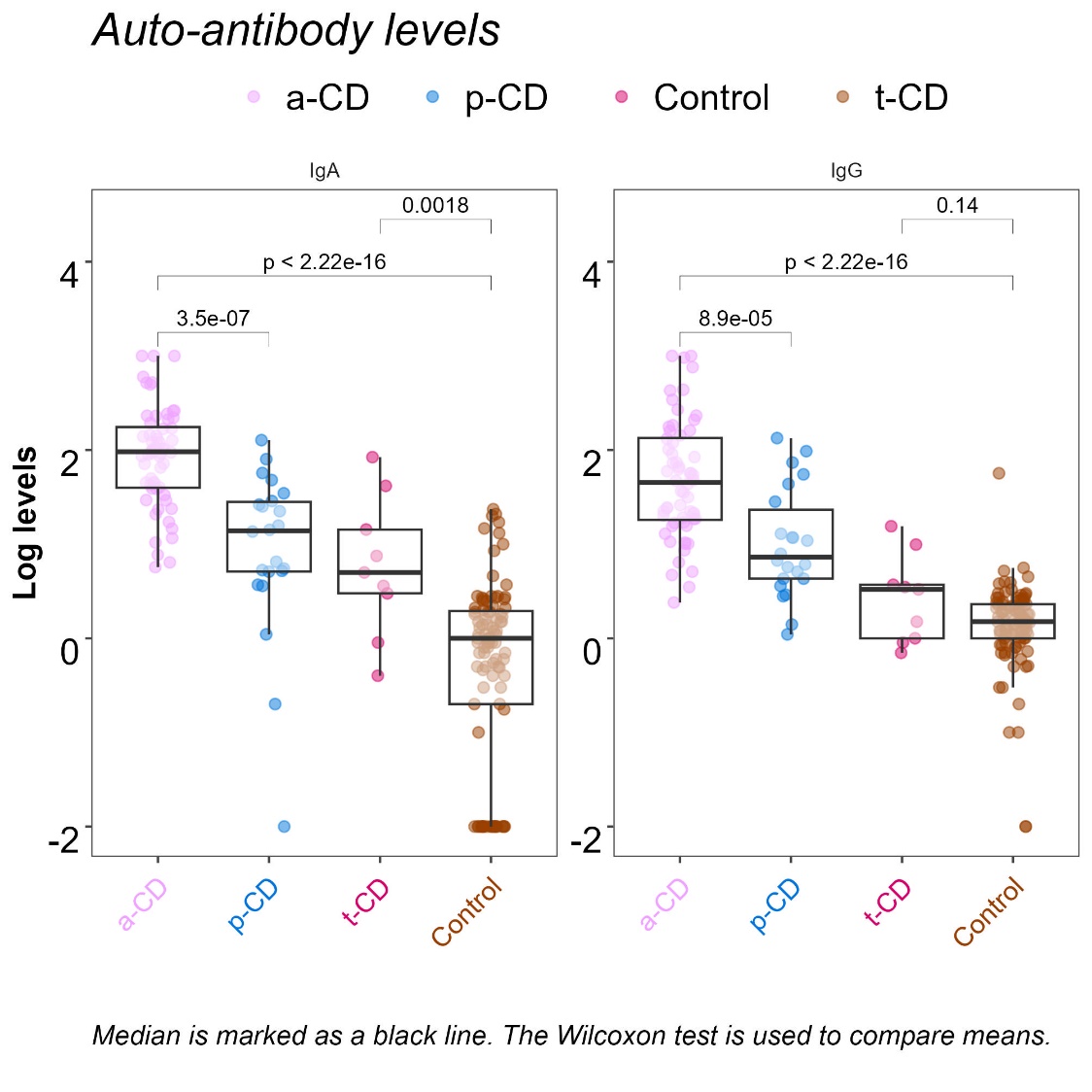


**Figure S2.** Bitter taste receptors (taste 2 receptors, TAS2Rs) levels in the different patient groups


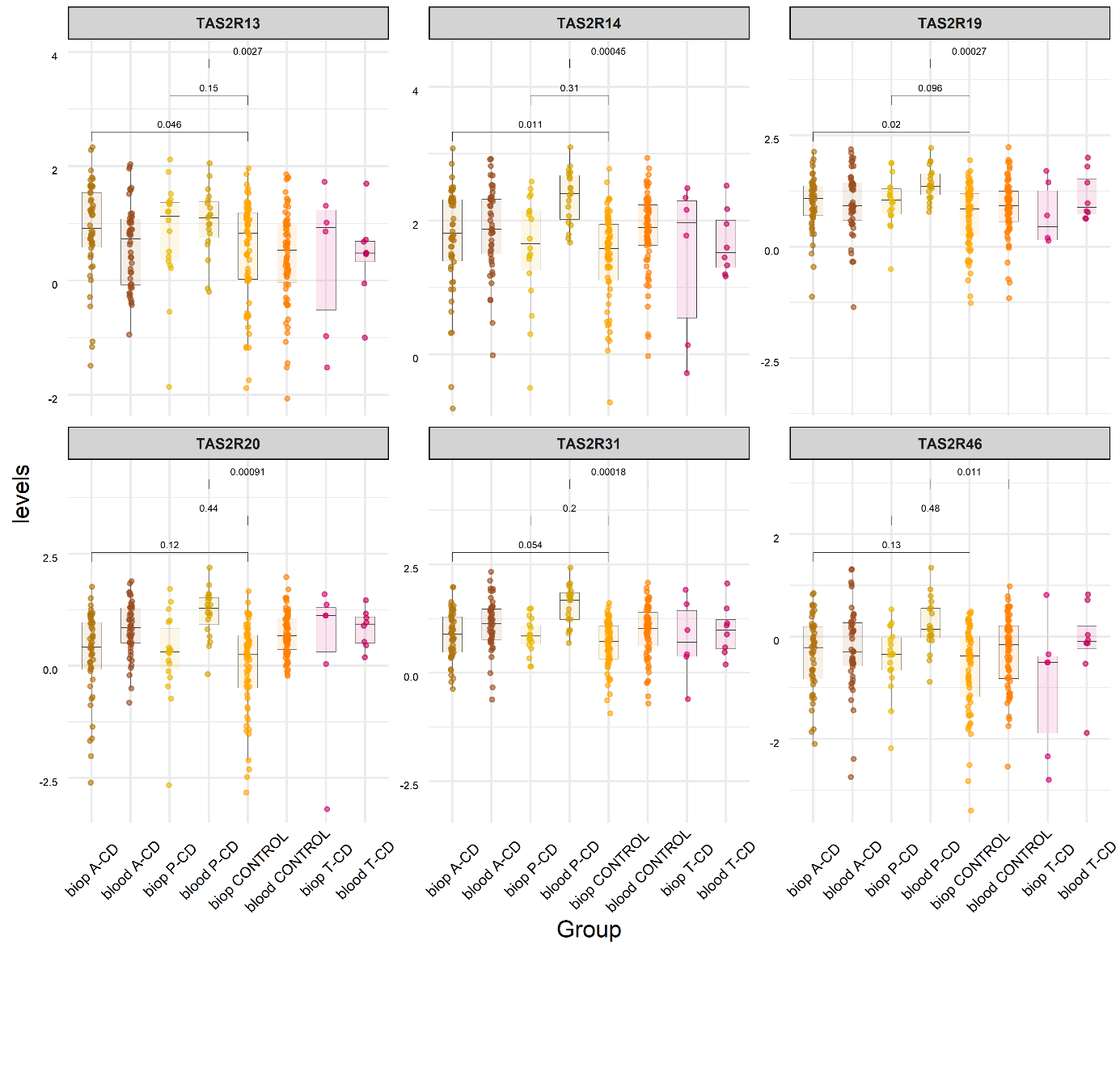

Supplement: Supplementary file 2 — Additional File 2: Table S2, Fig. S1-S2, Table S2. The top 1000 differentially expressed genes in small intestinal biopsies between patients with active celiac disease and controls, (a-CD vs CTRL) and the top 500 genes in comparisons including potential CD and treated CD (a-CD vs p-CD, a-CD vs t-CD, p-CD vs CTRL and t-CD vs CTRL). Figure S1. Tissue transglutaminase auto-antibody levels in the different patient groups Figure S2. Bitter taste receptors (taste 2 receptors, TAS2Rs) levels in the different patient groups. [file 12916_2025_4261_MOESM2_ESM.docx]
